# Predicting temperature-dependent transmission suitability of bluetongue virus in livestock

**DOI:** 10.1186/s13071-021-04826-y

**Published:** 2021-07-30

**Authors:** Fadoua El Moustaid, Zorian Thornton, Hani Slamani, Sadie J. Ryan, Leah R. Johnson

**Affiliations:** 1 Department of Biological Sciences, Virginia Tech, Blacksburg, VA 24061 USA; 2 Global Change Center, Virginia Tech, Blacksburg, VA 24061 USA; 3 Department of Statistics, Virginia Tech, Blacksburg, VA 24061 USA; 4 Computational Modeling and Data Analytics, Virginia Tech, Blacksburg, VA 24061 USA; 5 Computational Biology Program, Fred Hutchinson Cancer Research Center, Seattle, WA USA; 6 Department of Genome Sciences, University of Washington, Seattle, WA USA; 7 Quantitative Disease Ecology and Conservation (QDEC) Lab, Department of Geography, University of Florida, Gainesville, FL 32601 USA; 8 Emerging Pathogens Institute, University of Florida, Gainesville, FL 32610 USA; 9 School of Life Sciences, University of KwaZulu, Natal, South Africa

**Keywords:** Bluetongue virus, Vector-borne diseases, Transmission, Bayesian analysis, Temperature, Disease modeling

## Abstract

The transmission of vector-borne diseases is governed by complex factors including pathogen characteristics, vector–host interactions, and environmental conditions. Temperature is a major driver for many vector-borne diseases including Bluetongue viral (BTV) disease, a midge-borne febrile disease of ruminants, notably livestock, whose etiology ranges from mild or asymptomatic to rapidly fatal, thus threatening animal agriculture and the economy of affected countries. Using modeling tools, we seek to predict where the transmission can occur based on suitable temperatures for BTV. We fit thermal performance curves to temperature-sensitive midge life-history traits, using a Bayesian approach. We incorporate these curves into *S*(*T*), a transmission suitability metric derived from the disease’s basic reproductive number, $R_0.$ This suitability metric encompasses all components that are known to be temperature-dependent. We use trait responses for two species of key midge vectors, *Culicoides sonorensis* and *Culicoides variipennis* present in North America. Our results show that outbreaks of BTV are more likely between 15$^{\circ }$ C and $34^{\circ }\hbox { C}$, with predicted peak transmission risk at 26 $^\circ$ C. The greatest uncertainty in *S*(*T*) is associated with the following: the uncertainty in mortality and fecundity of midges near optimal temperature for transmission; midges’ probability of becoming infectious post-infection at the lower edge of the thermal range; and the biting rate together with vector competence at the higher edge of the thermal range. We compare three model formulations and show that incorporating thermal curves into all three leads to similar BTV risk predictions. To demonstrate the utility of this modeling approach, we created global suitability maps indicating the areas at high and long-term risk of BTV transmission, to assess risk and to anticipate potential locations of disease establishment.

**Supplementary Information:**

The online version contains supplementary material available at 10.1186/s13071-021-04826-y.

## Background

With ongoing climate change, it is critical that we understand how temperature influences the dynamics of emerging diseases. Vector-borne diseases (VBDs) are highly sensitive to climate factors, particularly temperature, as demonstrated previously for VBDs of both humans and plants [1–5]. Bluetongue virus (BTV), in the *Reoviridae* family (genus *Orbivirus*), causes bluetongue disease in livestock across the world and is thus a VBD of considerable economic concern. The biting midges of the *Culicoides* family are responsible for transmitting BTV and many other arboviruses. More than 1400 species of *Culicoides* have been classified globally, but fewer than 30 have been identified as competent vectors for BTV transmission [6]. These midges are highly sensitive to changes in temperature [7, 8], and thus so is BTV transmission [9, 10].

BTV can infect most species of domestic and wild ruminants, including sheep, goats, and cattle [11]. Sheep are the most susceptible to the disease and exhibit the highest morbidity and mortality post-infection [12, 13]. In the majority of infections by strains of BTV’s 27 serotypes, animals rarely show any clinical signs [14]. The infection severity and the presence of clinical signs both depend on the serotype, and the severity of infection can range from rapid death to quick recovery. Common outward clinical signs include a blue tongue, fever, and excessive salivation [13]. Since clinical signs are rare, BTV infection often goes without detection. Unfortunately, undetected cases can still result in mortality, and while BTV vaccines exist, vaccine development is in its infancy [15]. An effective polyvalent vaccine to immunize against more than one strain of BTV has yet to be developed [16], and existing attenuated viral vaccines pose significant health risks to livestock, such as reduced milk production in lactating sheep, abortion, early embryonic death, and teratogenesis in pregnant females [17].

**Fig. 1:**
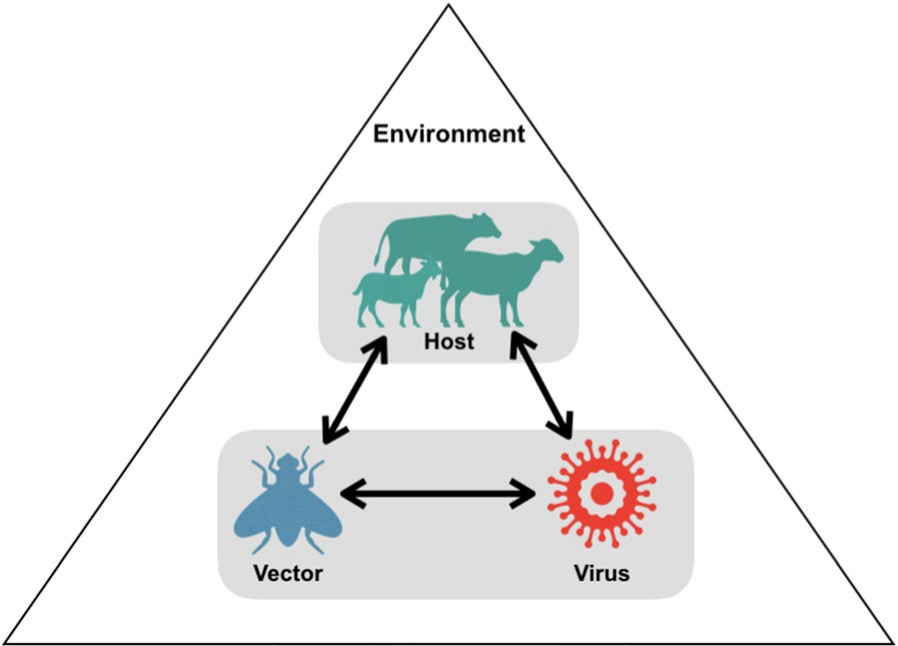
Bluetongue virus interaction diagram: the mechanisms underlying the transmission of bluetongue virus include host–vector interactions, host–pathogen interactions, and vector–pathogen interactions, as well as the environmental effect on all interactions

In the absence of an effective polyvalent BTV vaccine, and with the potential risks and costs of the available vaccines, the impact of BTV on global agriculture is significant. For example, the cost of BTV in the US beef industry was estimated at $95 billion in 2014 [16]. Although BTV was first detected among merino wool sheep in South Africa in 1905, since then the disease has been found on every continent but Antarctica [18]. In recent years, the disease has spread to areas previously believed to not be at risk, including Northern and Central Europe, parts of Asia, and Western North America [12, 19]. Mandatory testing of animals and losses in foreign markets impose a huge economic burden. This adds to the economic impact of BTV on the livestock industry. Substantial improvement is needed in our ability to assess risks and to anticipate potential shifts in risk over time and space.

Though the cause of the recent appearance of BTV in some of the new regions (especially Northern Europe) is still unknown, it is believed that climate change is a major driver. More specifically, the increase in temperature of certain locations makes them suitable for midges to survive, and therefore transmit diseases [13].

For example, some cases of BTV-8 in Europe, specifically in France, have exceeded expectations of receding and survived cold winters [20].

Mathematical modeling can facilitate our understanding of the complexities of the transmission process of vector-borne diseases [10, 21, 22]. The classical Ross–MacDonald model of VBDs and similar models allow us to calculate the basic reproductive ratio $R_0$ of the disease [23, 24]. This summary quantity is widely used to estimate how infectious a disease is and whether an outbreak can occur. When $R_0 >1$, the disease is likely to spread, leading to an outbreak; when $R_0 < 1$, the disease is likely to die out. As shown in Fig. 1, BTV transmission involves host–vector interactions, host–virus interactions, vector–virus interactions, and the effect of the environment. Mathematical models allow us to describe these interactions, parameterize them with data, and quantify the knock-on effects for transmission risk.

Here we are interested in answering the following questions: (1) How does the risk of transmission of BTV vary with temperature? (2) Do different model assumptions lead to different predicted suitability ranges? and (3) Which traits contribute the most to variation in estimates of transmission risk? To answer these questions, we take an approach used previously for VBDs such as malaria [1, 2]. We begin by using Bayesian inference to fit thermal responses to laboratory-derived data for temperature-sensitive midge life-history traits. We then derive $R_0$ for BTV as a function of these thermal responses and incorporate the fitted thermal responses to obtain estimates of these across temperatures. To focus on just the temperature-dependent components, we define a suitability metric, *S*(*T*), that isolates the temperature-sensitive components of $R_0$. We compare forms of *S*(*T*) where the midge density, *V*, is constant versus temperature-sensitive to ascertain whether this generates major differences in suitability predictions. Next, we conduct uncertainty analyses to identify which parameters drive uncertainty in *S*(*T*). This can indicate that either further data collection is needed to refine estimates, or that certain parameters have greater impacts on BTV disease transmission at different temperatures. Finally, we visualize predictions of the fitted suitability framework to explore which geographical areas might be suitable for transmission in the current native range of the midges, or if they become established elsewhere. Furthermore, understanding which temperature range results in $S(T)>0$ for given levels of other fixed parameters in our model may inform prevention and control strategies that target particular parameters (e.g., adult mortality rates via pesticide application).

## Methods

### Derivation of $R_0$ and *S*(*T*)

To predict the outbreak potential of BTV, several forms of the basic reproductive number $R_0$ have been developed [10, 21, 22]. The classical reproductive ratio for a generic VBD [1, 25] is given by

1 $$\begin{aligned} R_0 = \left( \dfrac{V \;bc\; a^2 }{d\; H\; \mu }e^{-\mu /\nu }\right) ^{1/2}\; \text {from \;[25]} \end{aligned}$$

where *V* is midge population density; *bc* is vector competence (the product of the probability that a midge can transmit the infection to an uninfected host, *b*, and the probability that a midge gets infected when biting an infected host, *c*); *a* is the per-midge biting rate; $\mu$ is the adult midge mortality rate; $\nu$ is the pathogen development rate ($\nu =1/EIP$ with *EIP* the extrinsic incubation period); *H* is host density, and *d* is infected host recovery rate. The model used to derive this version of $R_0$ is a system of delay differential equations that assumes no exposed class and that susceptible midges move to the infected class shortly after contact with an infected host. A similar scenario can be described using a system of ordinary differential equations while expressing the delay between the contact with the infected host and midges becoming infectious in terms of an exposed class. In this case, the reproductive number for the midge-borne viral disease (BTV) can be expressed as

2 $$\begin{aligned} R_0 = \left( \dfrac{V \;bc\; a^2 }{d\; H\; \mu }\dfrac{\nu }{\nu +\mu }\right) ^{1/2} \;\text {from\; [10]} \end{aligned}$$

This version of $R_0$ is a reduced version of a model that uses multiple types of host and multiple types of midge species as in [10, 21].

**Fig. 2:**
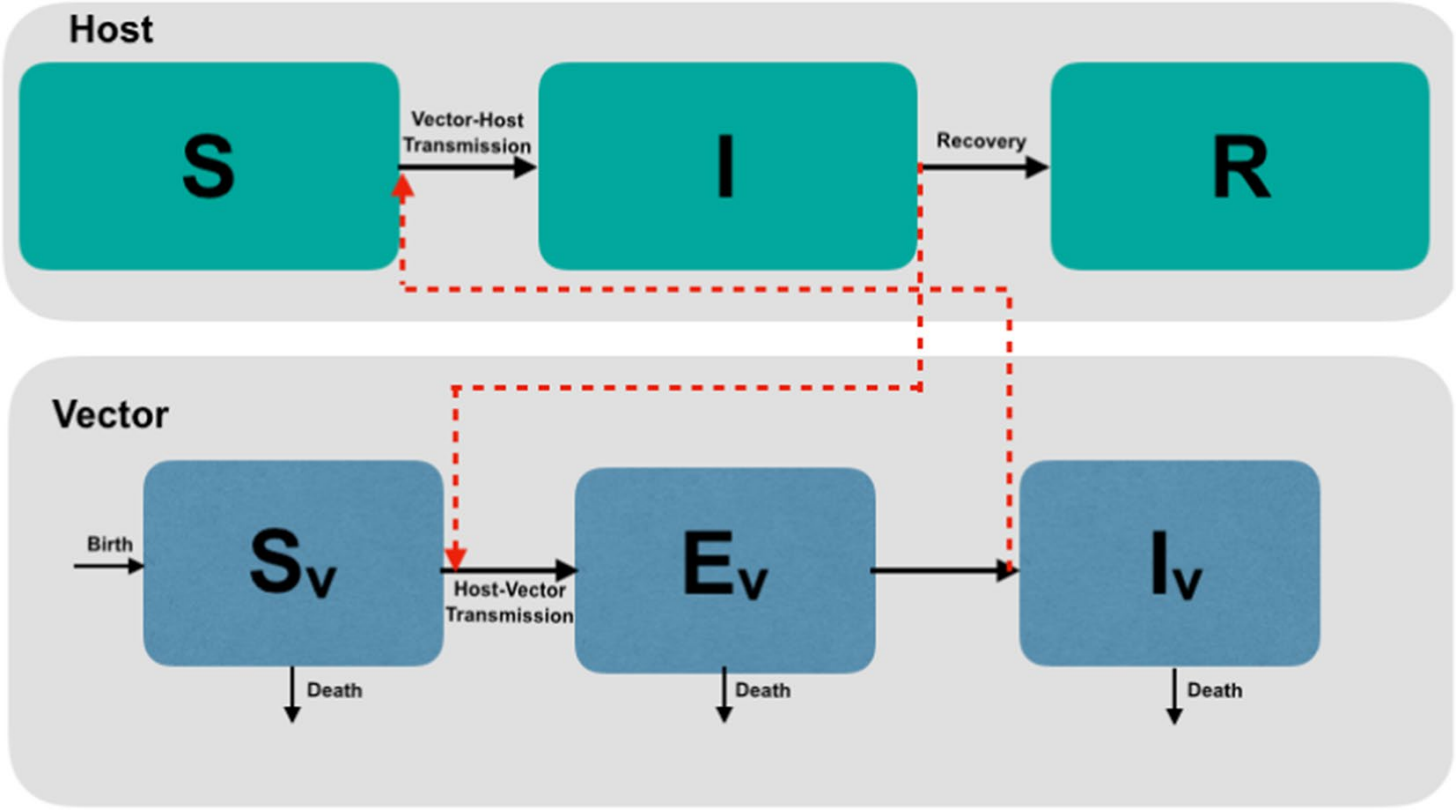
A schematic illustration of BTV transmission. The host population is composed of three classes: susceptible (*S*), infected (*I*), and recovered (*R*). The midge population is composed of a susceptible class ($S_v$), three exposed classes ($E_v$), and an infected class ($I_v$). Black arrows show movement between classes and red arrows indicate contact potentially leading to transmission

Figure 2 shows a schematic representation of our BTV transmission model (Equations A.1–A.8 in Additional file 1: Appendix A.1) which considers a single host population split into susceptible individuals that are vulnerable to BTV disease (*S*), infected individuals that have acquired infection (*I*), and individuals who have recovered from the disease (*R*). Also, we consider a vector population containing susceptible midges ($S_v$), three levels of exposed individuals ($E_v$), and an infected class of midges ($I_v$). The exposed classes in the model represent the extrinsic incubation period that midges undergo before becoming able to transmit infection. To calculate the third version of the basic reproductive number $R_0$, we use a next-generation matrix method described in [26, 27], which leads to the following $R_0$ equation:

3 $$\begin{aligned} R_0 = \left( \dfrac{V \;bc\; a^2 }{d\; H\; \mu }\left( \dfrac{3\;\nu }{3\;\nu +\mu }\right) ^3 \right) ^{1/2}. \end{aligned}$$

The term $\left( \dfrac{3\;\nu }{3\;\nu +\mu }\right) ^3$ in $R_0$ represents the number of midges that survive the extrinsic incubation period, leading to a slight difference between the three $R_0$ forms.

We can represent all three formulas of $R_0$ with a simple equation given by

4 $$\begin{aligned} R_0&= \left( \dfrac{V g f }{d\; H\; \mu }\right) ^{1/2} \end{aligned}$$

5 $$\begin{aligned}&= {\left( \dfrac{(midge\;density) \overbrace{(transmission\;potential)}^g \overbrace{(prob\; of\; becoming\; infectious)}^f }{(host\;recovery\; rate)\; (host\; density)\; (vector\;mortality)}\right) ^{1/2}} \end{aligned}$$

where the expressions for *V* and *g* are the same for all three versions and are given by

6 $$\begin{aligned} V&= \dfrac{F \;p_E\; p_L\; p_P}{\mu ^2\; (\rho _E + \rho _L + \rho _P)} \end{aligned}$$

7 $$\begin{aligned} g&= a^2\; bc \end{aligned}$$

where *F* is eggs per female per day, $p_E, p_L$, and $p_P$ are survival probabilities for eggs, larvae, and pupae; $\rho _E, \rho _L$, and $\rho _P$ are development times for eggs, larvae, and pupae, respectively; $\mu$ is adult midge mortality, *a* is midge biting rate, and *bc* is midge competence.

Although $R_0$ is a useful metric, particularly since the thresholding behavior can predict whether or not an epidemic can take hold, multiple factors, including the size of the susceptible population, whether or not parasites/hosts/vectors are physically present in an area, socioeconomic factors (e.g., screens, household and working conditions), or control measures, can all impact $R_0$ at a particular location. We want to focus our analysis strictly on the temperature components of the transmission, to be able to determine the temperatures that prohibit or promote transmission, and explore sensitivity to the thermal traits independently of other factors. Thus, we define a transmission suitability metric, *S*(*T*), as the (standardized) thermal components of $R_0$ (Eq. 4), and given by

8 $$\begin{aligned} S(T)&= C \left( \dfrac{V g f }{ \mu }\right) ^{1/2} \nonumber \\&= C\left( \dfrac{F \;p_E\; p_L\; p_P\;a^2\; bc\;f}{\mu ^3\; (\rho _E + \rho _L + \rho _P)}\right) ^{1/2} , \end{aligned},$$

where *C* is a constant that is chosen after the Bayesian fitting of traits (see below) that scales the median suitability to lie between 0 and 1. That is, we choose *C* to be the highest value of the posterior median suitability. When the median suitability is zero, this indicates that temperatures do not permit transmission, and when the median suitability is 1, this indicates a maximal transmission, everything else being equal.

The difference between the three $R_0$/*S*(*T*) formulas lies in the latent period survival probabilities, *f*, representing the probability of midges surviving to become infectious post-infection. Table 1 summarizes the latent period survival probabilities for each of the three models considered.

**Table 1:** Formulas for the probability of an infected midge (vector) surviving to become infectious, arising in $R_0$ formulas from different models, and the parameters involved

Formula	Traits used
$f_1=e^{-\mu \; \nu }$ [Dietz 1993]	$\mu :$ adult mortality rate
$f_2=\dfrac{\nu }{\nu +\mu }$ [Gubbins et. al. 2008 ]	$\nu :$ pathogen development rate
$$f_3=\left( \dfrac{3 \nu }{ 3 \nu +\mu }\right) ^3$$	

**Fig. 3:**
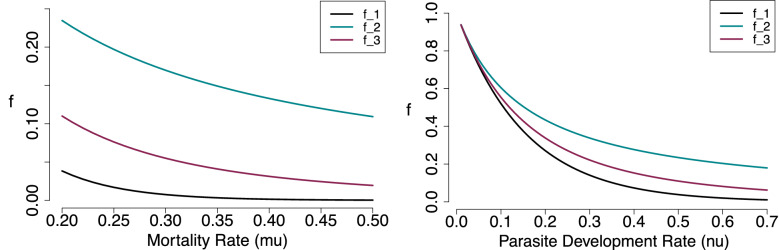
(Left) Latent period survival probability *f* versus midge mortality rate $\mu$ with a fixed $\nu = \mathrm {mean}(\nu (T))=0.061$. (Right) Latent period survival probability *f* versus pathogen development rate $\nu$ with a fixed $\mu = \mathrm {mean}(\mu (T)) = 0.15$

In Fig. 3, we plot all three latent period survival probabilities with one parameter fixed as the other varies (e.g., with virus development rate, $\nu$, fixed and midge mortality rate, $\mu$, varying). We use all three forms in our analysis while comparing the constant vector density case *V* to temperature-sensitive density *V*(*T*).

### Bayesian fitting of temperature-sensitive traits

As ectotherms, midges are sensitive to temperature. The thermal performance for these temperature-dependent traits is generally hump-shaped, starting at zero at a given minimum temperature, then increasing to a peak value as temperature increases, then sharply dropping to a lower value at a maximum temperature [28, 29]. However, depending on how a trait is measured, the pattern may instead be concave up. For example, mortality rates exhibit this pattern, such that the mortality is lowest at intermediate temperatures.

Here, we collected trait data corresponding to two midge species from the family *Culicoides*, namely, *Culicoides sonorensis* and *Culicoides variipennis*, both found in the United States [30]. The data collection method consisted of synthesizing data from the published literature by assembling data from tables and digitizing data points from graphs; details on data used for fitting for each trait are provided in  Additional file 1: Appendix A.4 and A.6. We focused on data from controlled laboratory experiments on midge trait variation at constant temperatures, ideally with three or more data points. For digitization, we used Plot Digitizer free software [31].

We used the temperature-dependent trait fits in all three $R_0$/*S*(*T*) formulations for comparison. Following a method first introduced in [1], we fit unimodal curves to temperature-sensitive traits. For the unimodal curves, we chose between a Brière curve (Eq. 9) for left-skewed data or a quadratic formula (Eq. 10) for symmetric traits.

9 $$\begin{aligned} \text {Bri}\grave{\text {e}}\text {re: } k T (T- T_{Min})\sqrt{T_{Max}-T} \end{aligned}$$

10 $$\begin{aligned} \text {Quadratic: } inter - n.slope\; T + qd \;T^2 \end{aligned}$$

where the constants *k*, $T_{Min}$, $T_{Max}$, *inter*, *n*.*slope*, and *qd* are estimated from trait data. For more information on the prior and fitted values, see Additional file 1: Appendix A.6

Similarly to [2], we used a Bayesian approach for our fitting method. For each continuous positive trait, we chose a truncated normal distribution as our likelihood. When fitting probabilities/proportions, we instead either used a binomial likelihood (when raw count data was available) or used a normal likelihood truncated at zero and one if only summarized data were available. We chose priors for thermal performance curve (TPC) parameters to ensure that parameters had biologically reasonable sign and range.

We used Markov chain Monte Carlo (MCMC) sampling in JAGS/rjags to fit our models [32]. For each trait, we ran five MCMC chains with 5000-step burn-in followed by 25,000 samples. Of these we kept every fifth sample, to obtain 5000 thinned samples for subsequent analyses. We used these 5000 samples of each parameter to calculate the associated trait thermal curves, resulting in 5000 thermal fits of the trait data. After generating the 5000 posterior mean curves for each trait, we used the 5000 posterior curves to generate posterior curves for $R_0$/*S*(*T*). For all posteriors (i.e., of traits and *S*(*T*)), we summarized posterior distributions using the temperature-dependent medians and the corresponding 95% highest posterior density (HPD) interval, which is the smallest credible interval in which 95% of the distribution lies [33]. All analyses were implemented in R [34]. More details on likelihoods and priors used can be found in Additional file 1: Appendix A.6.

### Uncertainty in *S*(*T*)

The *S*(*T*) formula (Eq. 8) depends on multiple temperature-sensitive traits, and so does its posterior density. Hence, there are many sources of uncertainty in the mean posterior density that can be identified through uncertainty analysis. We sought to isolate the contributions of each component of the model to the overall uncertainty through a variation on a traditional sensitivity analysis.

We calculated the uncertainty associated with *f*, *g*,  and *V* by varying one while keeping the rest fixed at their posterior means. We calculated the width of the 95% credible interval around the mean posterior curve, i.e. the difference between the upper and lower quantiles when only one of the components is allowed to vary. We then divided this by the width of the interval when all are allowed to vary. We repeated this process for each component *f*, *g*, and *V*, and then plotted all the curves together against temperature. This allowed us to identify which model component is responsible for the largest proportions of uncertainty in *S*(*T*) by identifying the curve with the highest value at a given temperature.

### Mapping suitability

The concern about climate-mediated increases and shifts in BTV risk is best visualized using mapping approaches, to understand where suitability is permitted and for how long, and how much livestock are thus at risk. Existing mapping approaches to this question largely focus on the European landscape, due to the recent uptick in BTV outbreaks. However, existing models purport to capture a general *Culicoides* spp. model but must rely on data from the UK vector *Culicoides obsoletus* mixed with other species that may not be the dominant vector, or even currently present. In this study, we focus on the two US vectors for which there are data and project a global risk. We do this under the assumption that given the capacity for *Culicoides* to spread and establish—as demonstrated by the Afrotropical *C. imicola* invasion across Southern Europe in recent decades—there may well be similar invasions and establishment by the two well-studied US vectors, and thus specific models will provide useful planning tools.

To visualize and apply our understanding of the thermal suitability of BTV, we mapped both suitability and risk at a global scale. First, we define suitable regions as those where the posterior median of the suitability metric *S*(*T*) >0. This is equivalent to finding the values where the posterior probability that $S(T)>0$ is 0.5. We note that here we use a scaled form of *S*(*T*) as described above. We present the geography of suitability across the globe by mapping the number of months of suitable temperatures for transmission based on the monthly average temperatures from the WorldClim dataset [35]. We use these average monthly temperatures as a means to describe seasonality at a global scale with climate products that are comparable between baseline (current temperatures) and future scenarios, to lay the groundwork for future investigations. The WorldClim data provide a trade-off between a spatial and temporal resolution that facilitates conducting calculations of risk across the globe.

Second, we map livestock at risk of transmission using the latest Food and Agriculture Organization of the United Nations (FAO) Gridded Livestock of the World (GLW3) data for 2010, which details global distributions of sheep, goats, cows, and others, at a 5-minute scale [36]. To create a visually accessible risk map, suitability was scaled 0–1, and this was multiplied by $log_{10}$(1 + livestock). Thus we create a scaled risk map, balancing the season length and livestock density, to emphasize areas of coincidence rather than simple suitability. In this case, we used the GLW3 sheep distribution [37] as the primary host at risk. This gridded product has values ranging from 0 to >340,000 sheep per pixel. All map calculations and manipulations were run in R using packages raster [38, 39], maptools [40], and Rgdal [41], following methods described in [42, 43].

## Results

### Temperature-dependence model components

Here we summarize the model components that depend on temperature and explain their role in the model.

#### Midge thermal traits

**Fig. 4:**
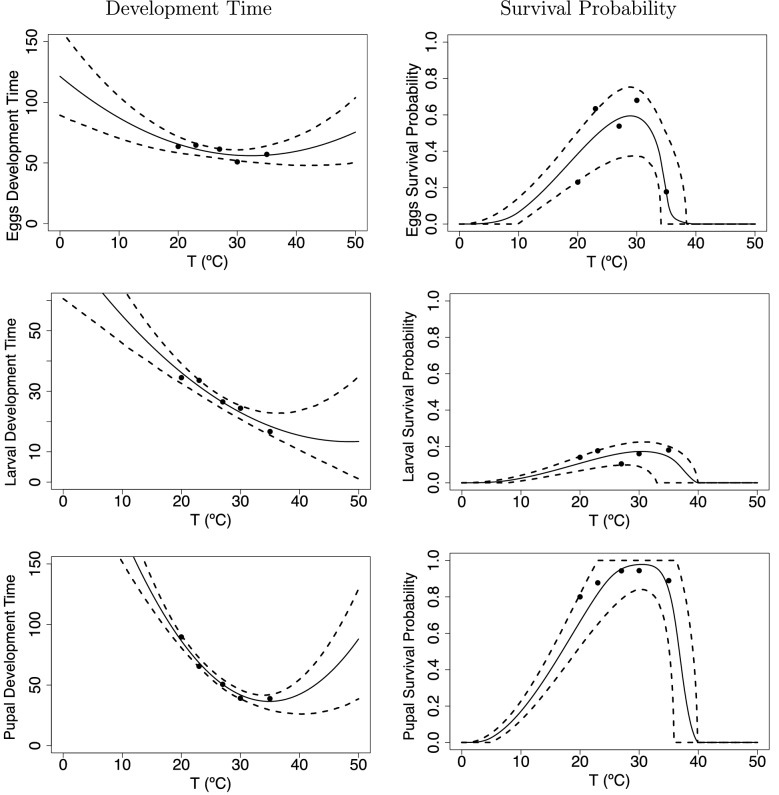
Figures in the left panels show development time in days for midge juvenile stages, eggs $\rho _E$, larvae $\rho _L$, and pupae $\rho _P$. Figures in in the right panels show survival probabilities for midge juvenile stages, eggs $p_E$, larvae $p_L$, and pupae $p_P$. The solid line is the mean of the posterior distributions of the thermal response curves, while the dashed lines represent the HPD intervals

In Fig. 4 we show data and fitted curves for development times and survival probability for eggs, larvae, and pupae. Development times (Fig. 4 left) are fitted assuming a quadratic function, under the assumption that juvenile midges at a given stage will need more time to develop at very low (<20 $^\circ$C) and very high (>35 $^\circ$C) temperatures. For eggs, the development time ranges from 60 to 70 days; for larvae, from 15 to 35 days; and for pupae between 40 and 80 days. We fit the survival probabilities using a Brière curve (Fig. 4 right). The survival probability is relatively high for eggs ($0.2<p_E<0.8$), very low for larvae ($p_L<0.2$), but almost always 100% for the pupae stage ($p_P \sim 1$).

**Fig. 5:**
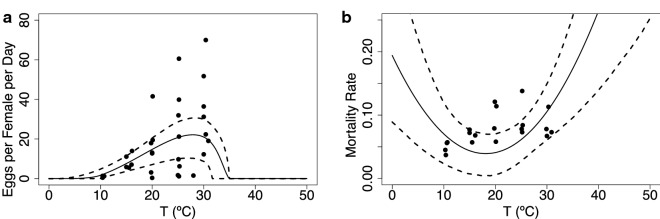
**a** Fecundity *F* (eggs per female per day) and **b** adult mortality rate $\mu$ traits as they vary with temperature. The solid line is the mean of the posterior distributions of the thermal response curves, while the dashed lines represent the HPD intervals

In Fig. 5a we show data on fecundity *F* (the number of eggs laid per female per day) together with the fitted Brière curve. The fecundity reaches a maximum at $\sim$ 30 $^\circ$C, and we do not have data for temperatures beyond that. The mortality rate, $\mu ,$ is fit using a quadratic curve where we assume that the mortality is highest for temperature less than 10 $^\circ$C and higher than 30 $^\circ$C (Fig. 5b).

**Fig. 6:**
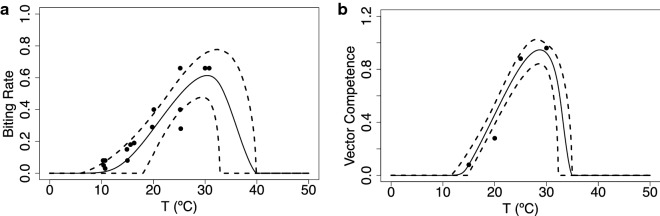
Biting rate (**a**) and probability that midges transmit infection when biting an uninfected host (**b**). The solid line is the mean of the posterior distributions of the thermal response curves while the dashed lines represent the HPD intervals

**Fig. 7:**
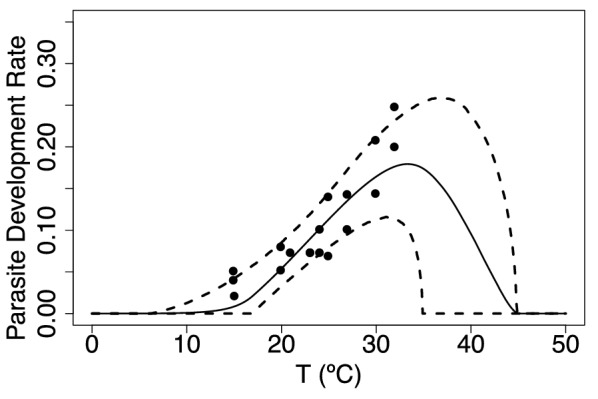
Virus development rate ($\nu$) is the inverse of the extrinsic incubation period ($\nu = 1/EIP$). The solid line is the mean of the posterior distributions of the thermal response curves, while the dashed lines represent the HPD intervals

In Fig. 6 we show the biting rate *a* and the transmission probability *b*, both fit with a Brière curve. The biting rate minimal values lie around 10 $^\circ$C, increasing to reach a maximum at 30 $^\circ$C, while the transmission probability *b* is lowest at around 15 $^\circ$C and reaches a maximum at 30 $^\circ$C. We do not have data for the infection probability *c*, so we assume that it is equal to 0.5. Lastly, Fig. 7 shows the virus development rate fit using a Brière curve, with minimal values around 15 $^\circ$C and maximal values around 32 $^\circ$C. Overall, these thermal traits all lack data values at extreme temperatures.

#### Midge density *V*

Recall the midge density formula given by

11 $$\begin{aligned} V(T) = \dfrac{F(T) \;p_E(T)\; p_L(T)\; p_P(T)}{\mu (T)^2\; (\rho _E(T) + \rho _L(T) + \rho _P(T))} \end{aligned}$$

**Fig. 8:**
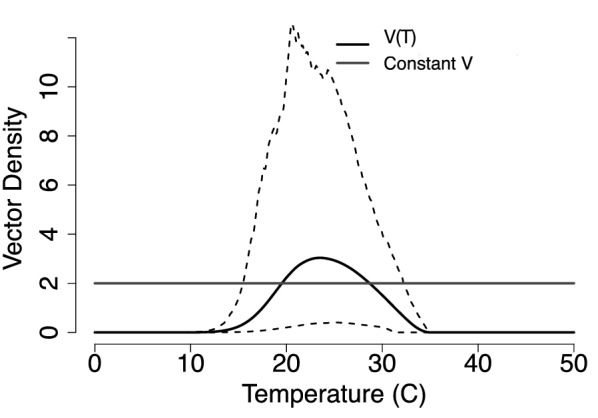
Modeled midge density as it varies with temperature. To obtain the temperature-dependent midge density, V(T), we evaluate Eq. 11 at all temperature-dependent traits using the fitted curves. The solid black line shows the estimated density, and the dashed lines show the corresponding HPD interval. A constant value $V=2$ is shown for comparison for subsequent modeling where the density is constant

To estimate midge density *V*, we use the posterior samples of the survival probabilities $p_E,\; p_L,\;p_P,$ for egg, larvae, and pupae; the development times $\rho _E,\;\rho _L,$ $\rho _P$ corresponding to the egg, larvae, and pupae life stages; the fecundity measure represented by the number of eggs per female per day *F*; and the adult mortality rate $\mu .$ In Fig. 8 we show that the posterior estimate of temperature-dependent midge density *V* is highest between 20 $^\circ$C and 28 $^\circ$C; it increases at temperatures higher than 10 $^\circ$C and decreases when the temperature exceeds 28 $^\circ$C.

#### Transmission potential

The component *g*, which we call the transmission potential, is estimated by calculating the product of midge biting rate *a* and vector competence *bc*:

12 $$\begin{aligned} g(T) = b(T)c \;a^2(T); \end{aligned}$$

**Fig. 9:**
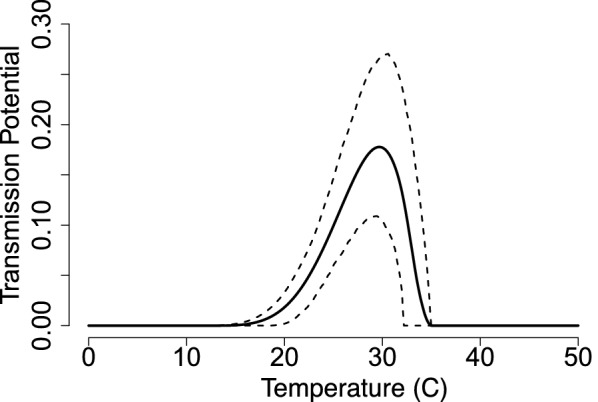
The transmission potential *g* as the biting rate *a* and transmission probability *b* vary with temperature while the infection probability is constant $c=0.5$. The solid line shows the estimated curve, and dashed lines are the HPD interval

Temperature-dependent data for transmission probability *c* were unavailable. Thus we assumed that there will be a 50% chance for midges to become infected after biting an infected host ($c = 0.5$) regardless of temperature. Figure 9 shows the posterior distribution of the predicted transmission potential thermal curve.

#### Functional form

**Fig. 10:**
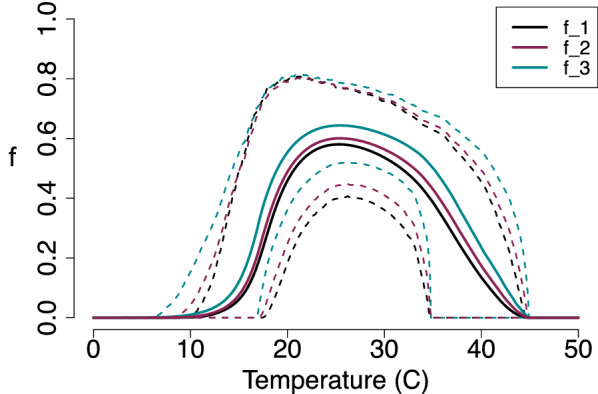
Latent period survival probability *f* used in $R_0$ versus temperature. The black line shows our model with the newly derived $R_0,$ the purple line shows the model presented in [10], and the blue line shows the model presented in [25]. Each solid line represents a different model, and the dashed lines show the corresponding HPD intervals. We note that there is an overlap between all HPD intervals, meaning that there are no statistically significant differences between these models

We explored three functional forms of the formula used to represent the probability of midges surviving to become infectious (Table 1). In all three cases, we calculate the thermal dependence of the functional form using the posterior distributions of mortality rate $\mu$ (Fig. 5b) and virus development rate $\nu$ (Fig. 7). Figure 10 shows the variation in the functional form with temperature based on the two temperature-dependent traits $\mu$ and $\nu$. Although there are differences in the magnitude of these curves, we can see that their peak occurs at the same temperature (25 $^\circ$C), which is due to the traits’ thermal dependencies. In addition, all of their HPD intervals overlap, which means that there are no significant differences between them.

### Thermal suitability *S*(*T*)

**Fig. 11:**
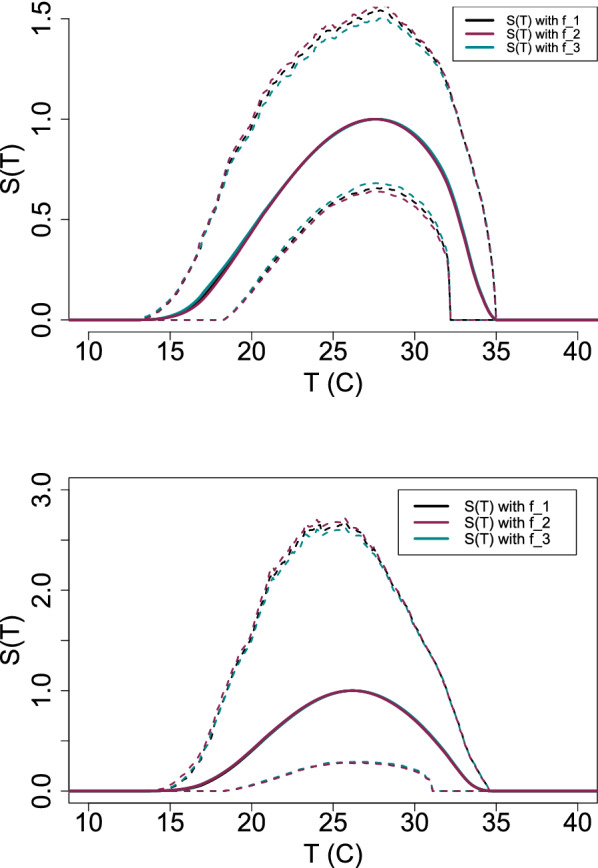
(Top) *S*(*T*) with constant midge density *V* and (Bottom) *S*(*T*) with temperature-dependent midge density *V*(*T*). The plots shows the magnitude of *S*(*T*) changing as temperature increases. Each solid line represents the mean of the posterior distributions of $R_0$, while the dashed lines are the HPD intervals

We use thermal traits to evaluate *S*(*T*) given by Eq. 8 with constant midge density *V* (Fig. 11 top) and with temperature-dependent midge density *V*(*T*) (Fig. 11 bottom). The three models are slightly different when constant midge density is used but are in agreement when temperature-dependent midge density is used. This is due to all the temperature-sensitive traits used to calculate *V*(*T*); however, this also leads to a higher uncertainty shown in the range of the HPD interval in Fig. 11 (bottom). The lower thermal bound of the three posterior means are different by a magnitude of 1 $^\circ$C. However, the peak temperature and upper thermal limits are in agreement for all three models. With these results, we predict that $S(T)>0$ occurs at a temperature greater than 15 $^\circ$C and less than 34 $^\circ$C, meaning that BTV is likely to cause an outbreak within this temperature range. We note that this prediction is based on assuming $c=0.5$, which may not always be true in reality.

### Source of uncertainty in *S*(*T*)

**Fig. 12:**
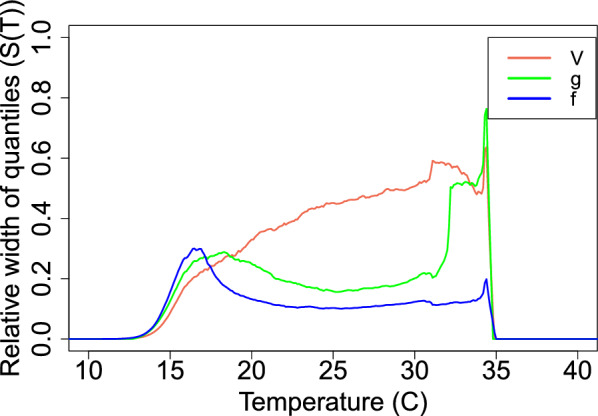
The source of uncertainty in *S*(*T*) is measured by calculating the relative width of the 95% HPD quantiles, with each component varying with temperature while the remaining components are kept constant, and divided by the width when all are allowed to vary

In Fig. 11 (bottom), a high variation around *S*(*T*) posterior density is shown in the large HPD interval. To determine the source of this uncertainty, we plot the calculated relative widths for each *S*(*T*) component, see Fig. 12. The results show that at a low-temperature range (14 °C <T< 18 °C), uncertainty in *S*(*T*) is mainly due to the uncertainty in the functional form *f*. At intermediate temperatures ($18\,^\circ C< T < 33\,^\circ C$), the uncertainty is caused by the midge density *V*(*T*). At very high temperatures ($33\,^\circ C< T < 35\,^\circ C$), the transmission potential *g* is the component producing the most variability in *S*(*T*).

### BTV risk maps

**Fig. 13:**
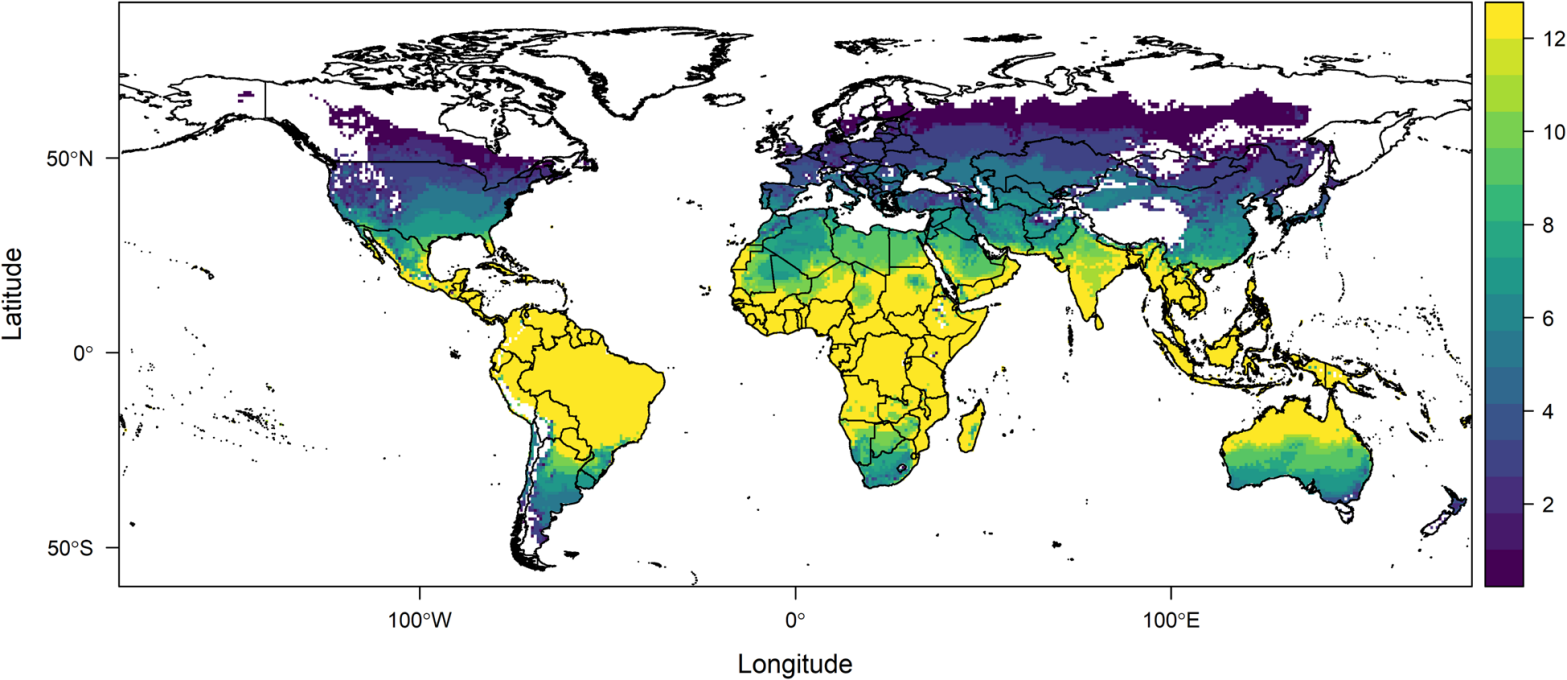
Map of the number of months (1–12) areas are at risk of bluetongue virus transmission according to our temperature-dependent $R_0$. This map is based on the current mean monthly temperatures and is restricted to bluetongue disease caused by the two midge species *Culicoides sonorensis* and *Culicoides variipennis*

Figure 13 illustrates the number of months each area is at risk of BTV transmission, with the assumption that *Culicoides sonorensis* and *Culicoides variipennis* are the main vectors. The results show that, under baseline long-term average current temperature conditions, much of Central Africa, South Asia, Central America, the northern part of South America, and northern Australia are suitable for year-round bluetongue transmission. These areas are also the warmest parts of the world, and as we move away from them, the temperature is lower and the number of months of suitability is reduced.

**Fig. 14:**
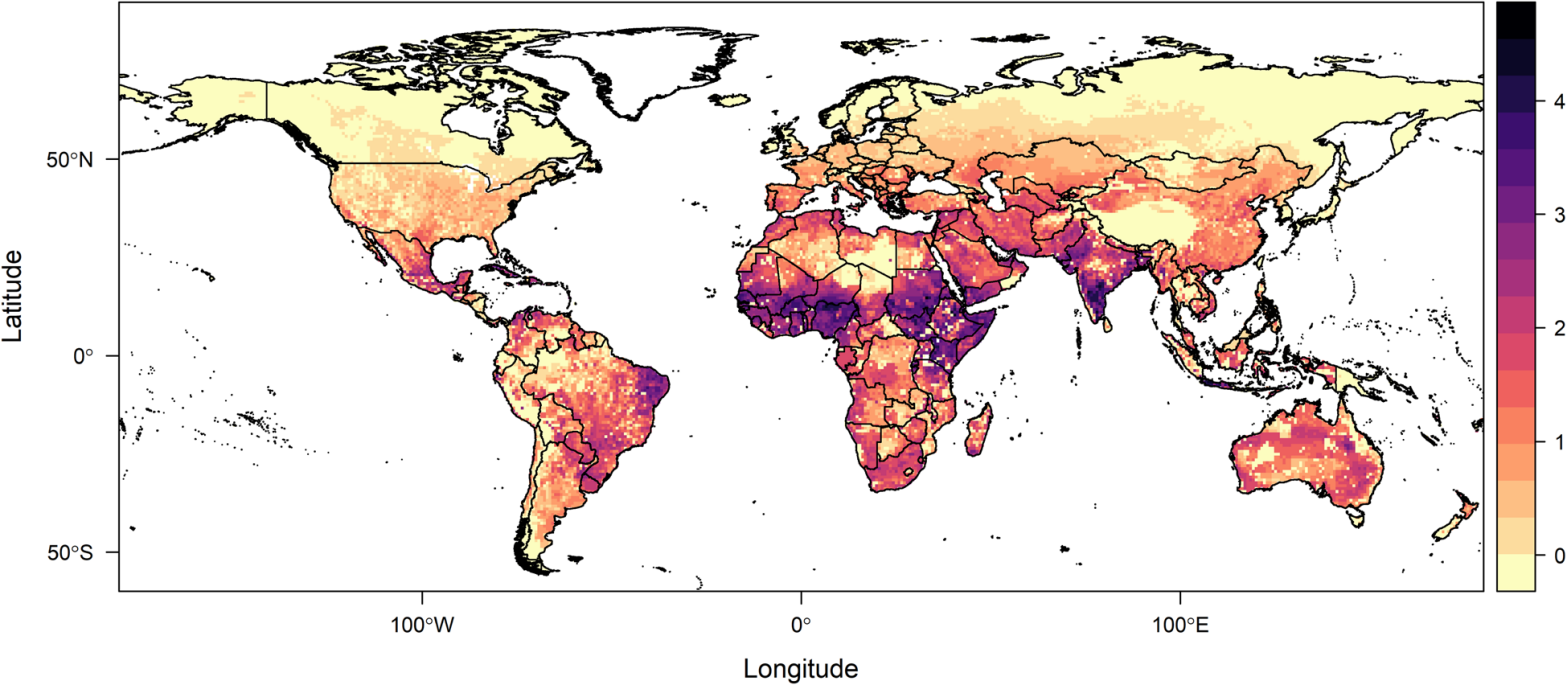
Scaled transmission risk suitability of bluetongue virus for sheep, as the primary host at risk, worldwide. The scale ranges from a low risk, 0, to a high risk, 5

Next, we used the global distribution of sheep to determine areas where sheep are at risk of acquiring BTV. The choice of sheep was mainly due to ready data availability, and also because sheep are the BTV host with the highest mortality and morbidity rates, and therefore of great interest and relevance. The map shows that areas where sheep are at the highest risk ($\text {scale}>3$) are located around the equator. The next highest risk regions ($1<\text {scale}<3$) are areas with high livestock industry, such as Central and South America and Europe (Fig. 14).

## Discussion

In this study, we are interested in the potential for the temperature to shape where BTV may spread. We use a Ross–Macdonald type modeling approach to describe the dynamics of BTV transmission [23, 24]. This mechanistic approach allowed us to derive the basic reproduction ratio’s posterior distribution as a function of temperature. We were able to determine both the suitable temperature for possible BTV outbreaks when $S(T) >0$ and the temperatures at which BTV outbreaks are likely to die out when $S(T) =0.$ We note that the absolute magnitude of the thermal response of *S*(*T*) here is dependent on our model assumptions, for example setting the infection probability to be $c=0.5.$ We also adopt two previously used BTV models, [25] and [10], to compare the three forms of $R_0$.

Based on the available trait data we used in our model, we predict that temperatures from 15 $^\circ$C to 34 $^\circ$C are “suitable” for BTV outbreaks by the examined midge species, with peak suitability occurring at about 26 $^\circ$C. This result was obtained regardless of which *S*(*T*) formula was used, i.e., all three different models of the latent period survival probability, *f*, led to the same predictions. Similarly, the predicted peak and upper thermal limit of *S*(*T*) were the same for the three forms, and only a small difference between lower thermal limits ($\sim$1 $^\circ$C) was observed. This indicates that the uncertainty of temperature effects on traits outweighs the effects of differences in modeling assumptions in the form of the latent period survival probability for these models. Because our suitability metric captures all of the temperature-dependent portions of $R_0$, this result should also hold for $R_0$ more broadly.

Uncertainty in temperature-dependent traits of the vector–virus system results in uncertainty in the suitability metric *S*(*T*). Our uncertainty analysis allowed us to determine the traits responsible for causing uncertainty in *S*(*T*) (and therefore in the temperature-dependent components of $R_0$) across the temperature range. At lower temperatures ($14\,^\circ C< T < 18\,^\circ C$) more data are needed for the parasite development rate, $\nu$, and mortality rate, $\mu$, to reduce this uncertainty in the latent period survival probability, *f*. At moderate temperatures ($18\,^\circ C< T < 34\,^\circ C$), the uncertainty in *S*(*T*) is caused by *V*, meaning that more data are needed in traits contributing to estimating the midge density. At very high temperatures ($34\,^\circ C< T < 35\,^\circ C$) we need more data on vector competence *bc* and biting rate *a*. Reducing the uncertainty in these components of *S*(*T*) will allow refinement of predictions, control, and prevention suggestions.

We were interested in using our derived suitability metric to determine areas at risk of BTV based primarily on temperature suitability. A risk map can be a useful planning tool, both to understand the scale of current risk and to anticipate suitable regions where the establishment of BTV could be successful were it to be introduced, with competent vectors. We created global risk maps showing the number of months per year each location worldwide is suitable for BTV disease transmission given the presence of two midge species, namely, *Culicoides sonorensis* and *Culicoides variipennis*. The results show that warmer areas are at risk year-round, while cooler areas are at risk for fewer months. Based on currently available data, few locations are predicted to have temperatures hot enough to exclude BTV for many months of the year. Further trait data to decrease the uncertainty near the thermal limits would enable more precise and accurate predictions. However, the particular predictions are also based on long-term, baseline current temperatures. With climate change, and the continuous rise in global temperatures, the area at risk of BTV may expand and shift to include places with previously lower risk, or some year-round locations may become too hot for year-round transmission [44, 45].

In building our maps, we chose to use monthly mean temperatures, as this captures the mean response of the suitability determined mechanistically. Other approaches might be to use climate products with different temporal resolutions and express suitability in the number of days between thresholds, but these products tend to be available at much coarser spatial resolutions, making them less suitable for combining with livestock layers. Instinctively, one may want to use minimum or maximum temperatures to impose thresholds, but this faces a very biological conundrum of model mechanics—a minimum or maximum temperature may exist for a very small time period within a given month, and thus not represent a longer period experienced by the vector in question. The behavioral avoidance mechanisms vectors can use in short periods of extremes would be missed by this approach, leading to underestimates of the potential extent of suitability.

Previous studies have investigated temperatures suitable for other vector-borne diseases. For example, a study on three mosquito-borne diseases, Zika, dengue, and chikungunya, transmitted by *Aedes aegypti* and *Aedes albopictus* showed that transmission is likely to occur between 18 and 34 $^\circ$C, with peak transmission between 26 and 29 $^\circ$C [43, 46, 47]. Moreover, the temperatures suitable for the transmission of the plant-borne disease, citrus greening, are between 16 $^\circ$C and 33 $^\circ$C, with peak transmission at 25 $^\circ$C [48]. Together with our findings, this shows that there are similarities between ectotherm vectors in the way they respond to temperature. For example, their traits follow hump-shaped thermal performance curves. But there are differences in the temperature ranges they tolerate, and the temperatures at which their performance is maximal. This points to the importance of building system-specific models for predicting the effect of extrinsic factors on the spread of VBDs.

As highlighted in a 2018 systematic review [49], BTV has been studied using many different modeling approaches. The systematic review summarized BTV models used post-1998 [49], most of which relied more on strong modeling assumptions than data. The model results were used to inform animal health decision-making by identifying at-risk areas and the risk of spread in the case of introduction [50] and climate change [45]. While several examined $R_0$ for BTV [10, 22], our model differs in that it incorporates temperature across a wide range, allowing us to estimate an $R_0$ that is also temperature-dependent. A more recent study used a mathematical quantity called vectorial capacity instead of $R_0$ to estimate BTV transmission [51]. $R_0$ and vectorial capacity are very similar, with the latter assuming perfect competence and ignoring host recovery rates (making our suitability metric somewhere in between). The study identifies the optimal transmission suitability range for *C. sonorensis* to be between 27 and 30 $^\circ$C, which overlaps with our transmission peak range of 26 and 29 $^\circ$C. The difference is likely due to our study including trait data for two *Culicoides spp.* as well as including temperature-dependent infection parameters. Overall, the two models are in agreement regarding the effects of gross temperature patterns on BTV transmission.

In addition, while data on *Culicoides spp.* temperature-dependent traits are scarce, we had the luxury of obtaining sufficient data to create a model for two North American vectors, and did not mix traits across species from different continents. This is of particular interest in assessing the potential for invasion and establishment (and hence spread) of disease vectors, which has been found to be almost a hallmark of *Culicoides spp.* across the European landscape in recent decades, leading to novel outbreaks of BTV. Linking $R_0$ or *S*(*T*) to temperature can help identify BTV outbreak risk based on the temperature at particular locations, which in turn can inform management policies and control strategies within current and changing climate conditions. By establishing a model specific to current vectors in the United States, we can assess the potential for invasion and spread to other parts of the globe.

## Supplementary Information

**Additional file 1.** Supplemental methods, figures, and tables.

## Data Availability

All data generated or analyzed during this study are included in this published article and its additional file.
