# Supplementary material for: Predicting temperature-dependent transmission suitability of bluetongue virus in livestock
Source: Parasit Vectors. 2021 Jul 30;14:382. doi: 10.1186/s13071-021-04826-y (PMC8323090; doi:10.1186/s13071-021-04826-y)
Supplement: Supplementary file 1 — Additional file 1. Supplemental methods, figures, and tables. [file 13071_2021_4826_MOESM1_ESM.pdf]

Additional File 1: Supplemental methods, figures, and tables for Predicting temperature-dependent transmission suitability of bluetongue virus in livestock

Fadoua El Moustaid[1,4], Zorian Thronton[2,3], Hani Slamani[2,3], Sadie J. Ryan[5, 6, 7], and Leah R. Johnson[1,2,3,4, *]

[1]Department of Biological Sciences, Virginia Tech, Blacksburg, VA 24061, USA

[2]Department of Statistics, Virginia Tech, Blacksburg, VA 24061, USA

[3]Computational Modeling and Data Analytics, Virginia Tech, Blacksburg, VA 24061, USA

[4]Global Change Center, Virginia Tech, Blacksburg, VA 24061, USA

[5]Quantitative Disease Ecology and Conservation (QDEC) Lab, Department of Geography, University of Florida, Gainesville, FL 32601

[6]Emerging Pathogens Institute, University of Florida, Gainesville, FL 32610

[7]School of Life Sciences, University of KwaZulu, Natal, South Africa

[*]Corresponding author: lrjohn@vt.edu

A Appendix

A.1 Transmission model for BTV

We use an SIR-SEI type of compartmental model to describe vector-host interactions in transmitting BTV (see Figure 2 in main text). The host population (H) is divided into susceptible (S), infected (I), and recovered (or immune) (R) classes, while the vector population (V) is divided into susceptible (S_V) and infected (I_V) classes as well as three Exposed (E_V) classes. Here we use three exposed classes in the vector population to incorporate a more realistic length of the extrinsic incubation period. Using three compartments with the exit rate from each compartment being 3 ν, lead to a Gamma distribution for overall midge progression to the infectious class with a mean rate of ν. Increasing the number of compartments used from 3 to a larger number leads to a Gamma distribution with lower variance around the mean [1]. This approach is an alternative to using fixed time delays, which are not suitable when using temperature-dependent parameters. Both host (H) and vector (V) populations are assumed (and are by definition of the model) constant.

$$\frac{dS}{dt} = -\frac{ab}{H} I_V S \tag{A.1}$$

$$\frac{dI}{dt} = \frac{ab}{H} I_V(t-\tau)S(t-\tau) - dI \tag{A.2}$$

$$\frac{dR}{dt} = dI \tag{A.3}$$

$$\frac{dS_V}{dt} = rV - \frac{ac}{H} I S_V - \mu S_V \tag{A.4}$$

$$\frac{dE_{V1}}{dt} = \frac{ac}{H} I S_V - 3\nu E_{V1} - \mu E_{V1} \tag{A.5}$$

$$\frac{dE_{V2}}{dt} = 3\nu E_{V1} - 3\nu E_{V2} - \mu E_{V2} \tag{A.6}$$

$$\frac{dE_{V3}}{dt} = 3\nu E_{V2} - 3\nu E_{V3} - \mu E_{V3} \tag{A.7}$$

$$\frac{dI_V}{dt} = 3\nu E_{V3} - \mu I_V. \tag{A.8}$$

where

$$H = S + I + R \tag{A.9}$$

$$V = S_V + E_{V1} + E_{V2} + E_{V3} + I_V \tag{A.10}$$

The model's parameters are presented in the Table A.1 below. Note that the parameters

Parameter	Definition	Units
d	Recovery rate of infected hosts	1/day
τ	Host's exposure period	day
r	Vector population's birth rate	1/day
a	Vector biting rate	bites / day
b	Probability that a midge is infected	dimensionless
c	Probability that a midge is infectious	dimensionless
μ	Mortality rate of adult vectors	1/day
ν	Parasite's development rate	1/day

Table A.1: Parameters used in the mathematical model, their description, and units.

τ is the time that a susceptible (S) takes to become infected after receiving a bite from infected vector (I_V). The parameter ν is the inverse of the Extrinsic Incubation Period of the pathogen (EIP), i.e., $\nu = \frac{1}{EIP}$. We define the vector population size to be $V = \frac{\lambda}{\mu}$, where λ is the total birth rate of adult midges in the whole population (adults/day), and μ is per-capita adult mortality rate (1/day). This is based on Parham & Michael [2], who derive the expression phenomenologically by treating V as a random variable. Thus, λ is equivalent to rV in the model above, given that $r = \mu$ at disease free equilibrium, and is given by

$$\lambda = \frac{F\, p_E\, p_L\, p_P}{(\rho_E + \rho_L + \rho_P)\, \mu} \tag{A.11}$$

where F is the number of eggs produced by all females in the population per day, $p_{E,L,P}$ are the survival probabilities in the Eggs, Larvae, and Pupae stages, and ρ_E, ρ_L, ρ_P are the development time in each stage. Then, the abundance of the vector becomes,

$$V = \frac{\lambda}{\mu} = \frac{F\, p_E\, p_L\, p_P}{(\rho_E + \rho_L + \rho_P)\, \mu^2} \tag{A.12}$$

A.2 Host recovery rate d sensitivity analysis

Although all ruminants are susceptible to BTV disease, each responds to the infection differently, with sheep being the most susceptible and showing extreme morbidity and mortality. In addition, BTV host recovery depends on the intensity of the infection as well as the time of disease detection, which results in recovery rate variability among hosts. To account for this, we perform a sensitivity analysis on the host recovery rate d by looking at the derivative of R_0 with respect to d as follows:

$$\frac{\partial R_0}{\partial d} = \frac{1}{2}\left(-\frac{V\ g\ f}{d^2\ H\ \mu}\right)\left(\frac{V\ g\ f}{d\ H\ \mu}\right)^{-1/2} = -\frac{1}{2d}\left(R_0^2\right)\left(R_0\right)^{-1} = -\frac{R_0}{2d}. \tag{A.13}$$

Since $\frac{\partial R_0}{\partial d} < 0$ always, the basic reproductive ratio R_0 increases as the recovery rate d decreases. Figure A.1 shows different R_0 densities corresponding to different host recovery rate values. Higher lengths of infection $1/d$, i.e. lower recovery rates d, are associated with higher R_0 densities, meaning that hosts with low recovery rate such as sheep are more challenging to manage as the chance of outbreak for them is more likely.

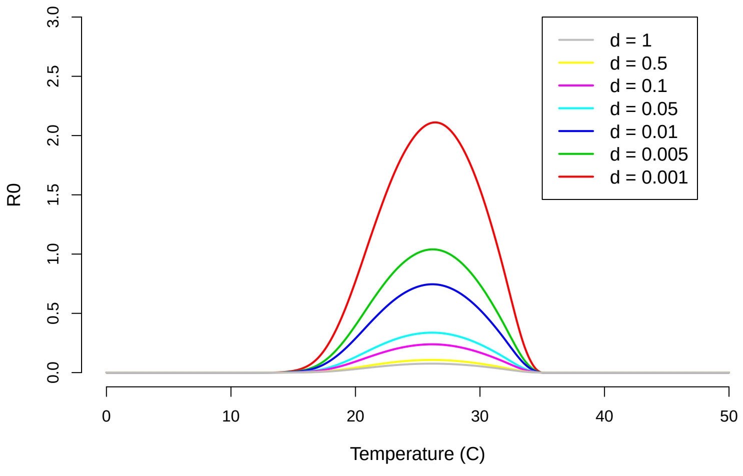

Figure A.1: Host recovery rate d values correspond to different R_0 posterior densities. As d decreases, R_0 density increases meaning that a lower recovery rate correspond to a higher outbreak risk.

A.3 Uncertainty analysis

We investigate the uncertainty caused in each of R_0 components, the midge density V, the functional form f, and the transmission potential g by examining the source of uncertainty within each component. For the midge density, the uncertainty is mainly caused by the adult midge mortality rate μ within a wide temperature range, from 10°C to 32°C. At higher temperatures (¿32°C) the uncertainty is caused by the fecundity F.

In the functional form case, the uncertainty is caused by the adult mortality rate μ for temperatures between 18°C and 32°C, this range overlaps with that of the midge density. At lower (10-18°C) and higher (32-45°C) temperature ranges, the uncertainty is caused by the pathogen development rate ν. In the transmission potential the overall uncertainty is caused by the biting rate a.

A.4 Bayesian fitting of traits thermal curves

To fit each trait, we chose a unimodal functional form as the mean function. We use normal distributions for most of the data while binomial distributions are used when fitting probability distributions. We used uninformative priors appropriate for the biological description of the data, taking into account the positivity of their values as well as their range. The values in the priors are decided as we go until the appropriate fitting curve is obtained.

Midges biting rate a

The biting rate of adult midges is one of many factors that influence Bluetongue transmission [3]. In order to calculate the biting rate, the time required for female *Culicoides sonorensis* to lay eggs after a blood meal, also known as a gonotrophic period, is required. Biting rate (a) can be approximated by taking the inverse of the gonotrophic cycle duration. Similar to other traits, the biting rate is sensitive to environmental factors, especially, temperature [3] (see Figure A.3 for thermal fit).

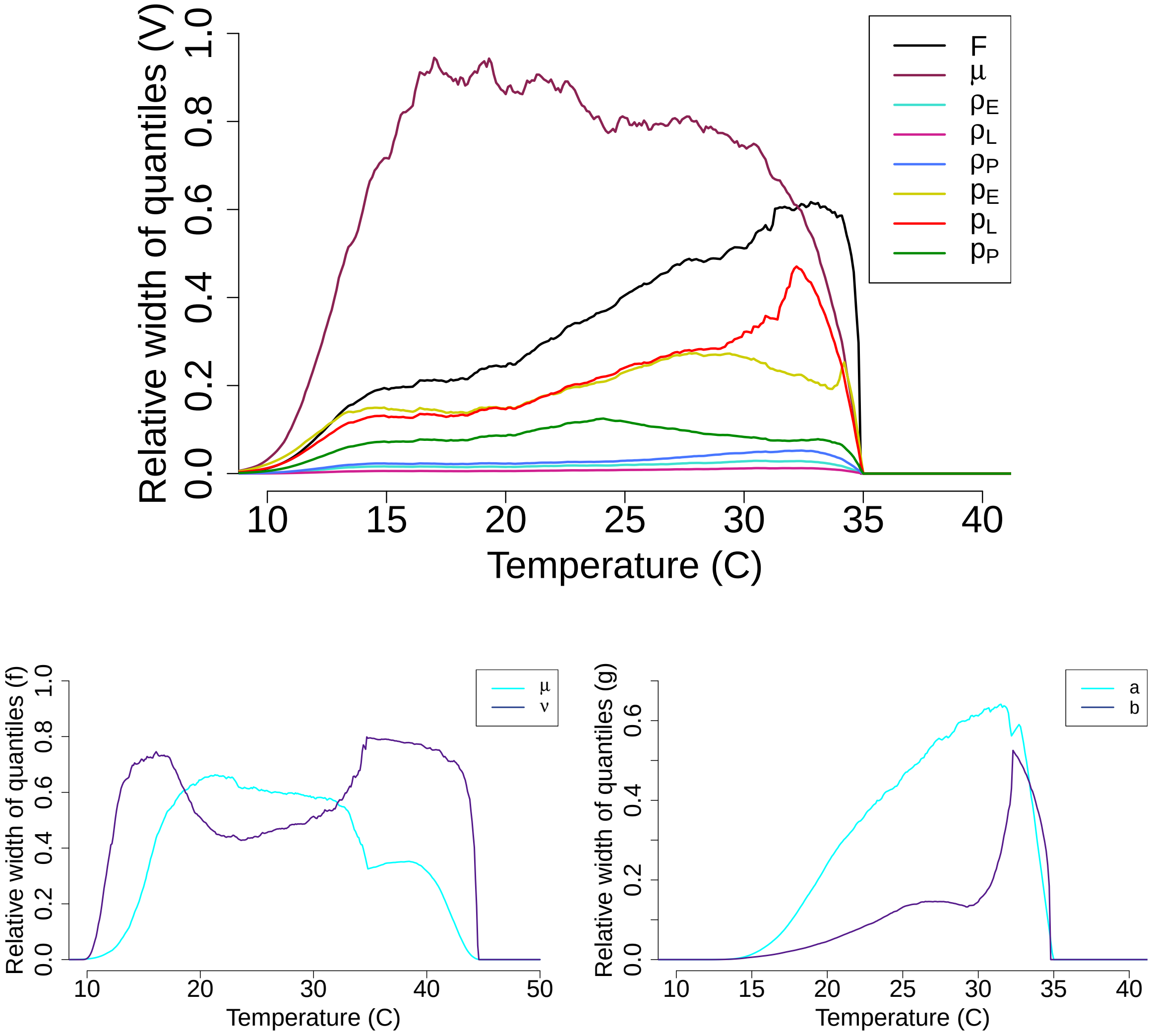

Figure A.2: The source of uncertainty in the midge density V (Top), the functional form f (Bottom-left), and the transmission potential g (Bottom-right) is measured by calculating the relative width of quantiles with each parameter varying with temperature while the remaining parameters are kept constants.

Vector competence bc

Vector competence for adult midges is a measure of their ability to transmit the disease. It is genetically determined and heavily influenced by environmental factors such as temperature and humidity [4]. Vector competence (bc) is the product of the probability of a vector getting infected after a blood meal containing a pathogen (c) and the probability of a vector transmiting infection (b). While we were able to find data for b concerning *Culicoides*

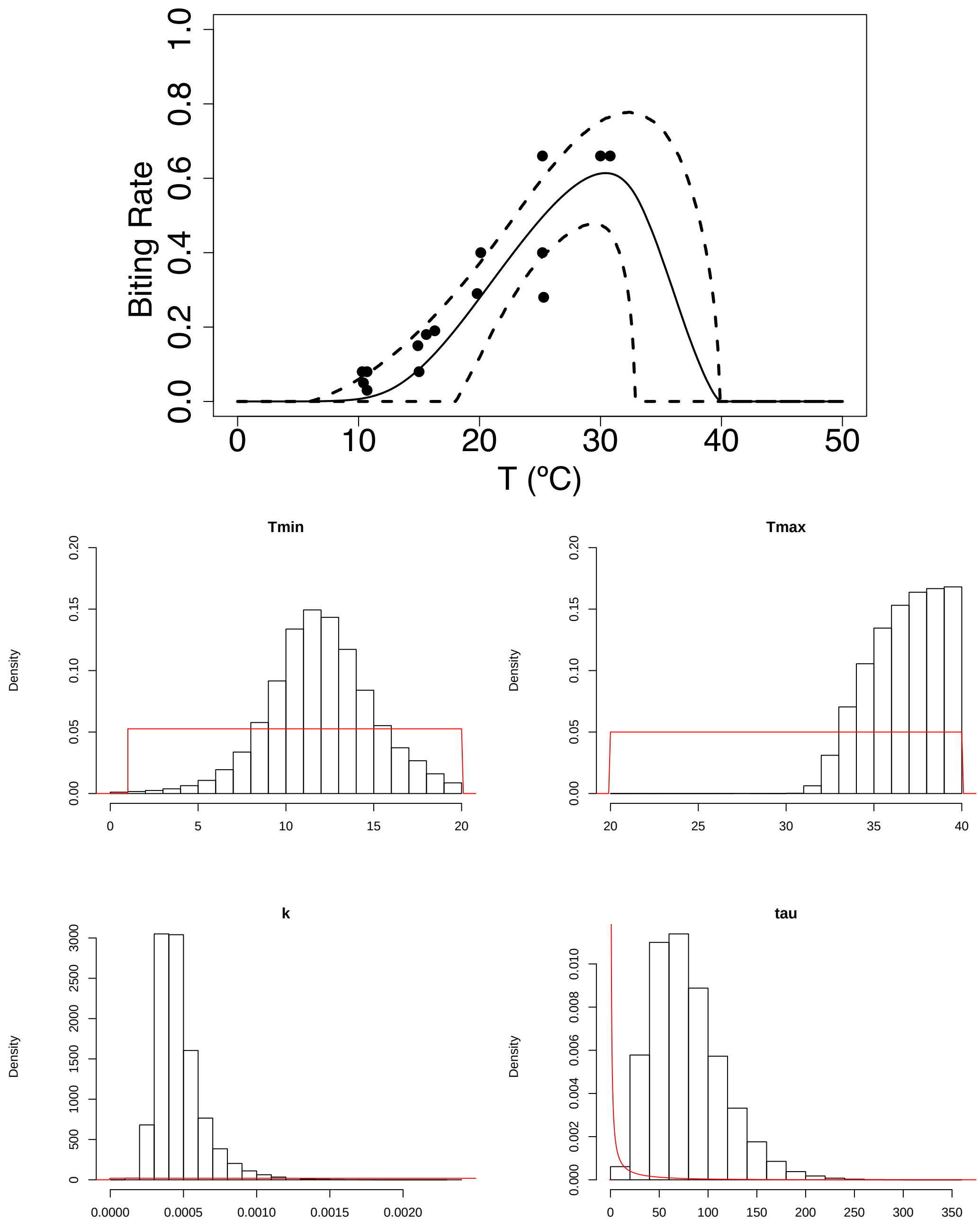

Figure A.3: (Top) The mean trajectory in solid line and HPD interval in dashed black for the biting rate a. (Bottom) Histograms of the posterior distribution for each parameter of the Brière fit for the biting rate a. The prior distribution for each parameter is plotted in red. The Brière fit is determined by the equation $kT(T - T_{Min})\sqrt{T_{Max} - T}$ using a normal distribution with precision τ.

sonorensis [5], we were unable to find data for c. We assume $c = 0.5$ for all calculations used in this analysis. We did fit a Bayesian model to the parameter b (Figure A.4).

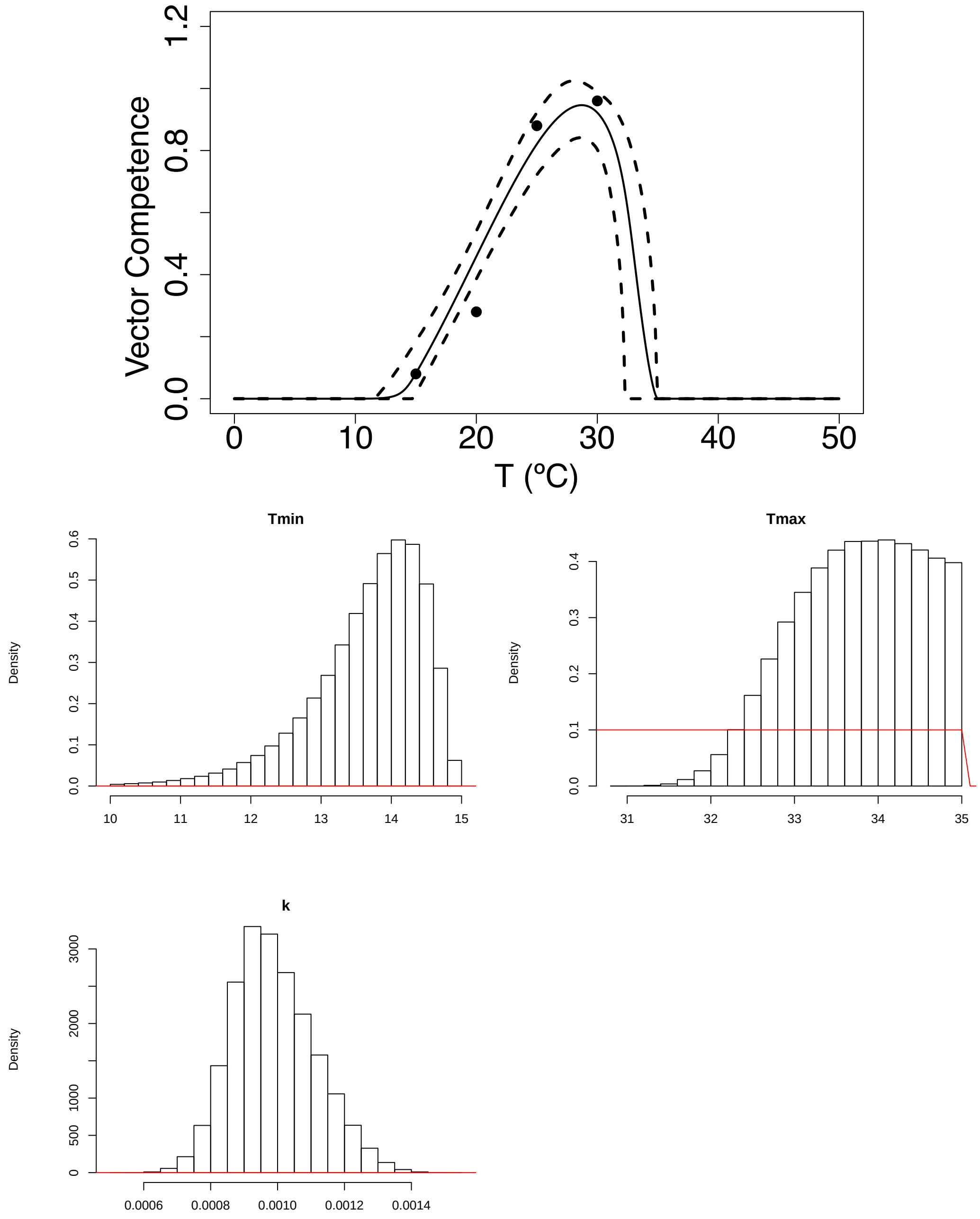

Figure A.4: (Top) The mean trajectory in solid line and HPD interval in dashed black for the probability of a vector transmitting the virus when biting b. (Bottom) Histograms of the posterior distribution for each parameter of the Brière fit for the probability b. The prior distribution for each parameter is plotted in red. The Brière fit is determined by the equation $kT(T - T_{Min})\sqrt{T_{Max} - T}$ using a binomial distribution.

Juvenile survival probability p_E, p_L, p_P

Vaughan et. al. studied the sub-adult life cycle of *Culicoides variipennis* at temperatures of 20 °C, 25 °C, and 28 °C [6]. We define the probability of an egg hatching by using the

mean percentage of laid eggs that hatched at each given temperature. We now define the probability of successful larval pupation by collecting the percentage of larva that ended up pupating at each given temperature. We finally define the probability of pupae emerging to become adults, p_P, as the mean percentage of pupae that survive to the adult stage at each given temperature (Figures A.5, A.6, A.7).

Juvenile development time ρ_E, ρ_L, ρ_P

Egg Development Time is defined as the time in days required for eggs to hatch in a given temperature. *Culicoides variiennis* were studied in a laboratory setting [6]. Larva Development Time is defined as the time in days required for the larva to mature into a pupa in a given temperature. Pupa Development Time is defined as the time in days required for a pupa to mature into adult midges in a given temperature (Figures A.8, A.9, A.10).

Fecundity F

The rate at which female midges lay eggs is closely related to the spread of Bluetongue. This rate is typically measured as eggs per female per day. For this study we also utilized fecundity data that was taken over two oviposition cycles and transformed the data (originally eggs per female) by dividing by the median oviposition time [3].

Pathogen development rate ν

Parasite development has been shown to increase with temperature in studies that support the hypothesis that global warming has been cause for latitudinal shifts which in turn increase the reach of vectors that transmit diseases like bluetongue [7]. In order to investigate this trait's relationship with temperature, we made use of data on Extrinsic Incubation Period (EIP) to create a new parameter: Parasite Development Rate (ν) ($\nu = 1/\text{EIP}$). EIP is the time between a vector getting infected with a pathogen to the time that the vector itself is able to transmit the pathogen.

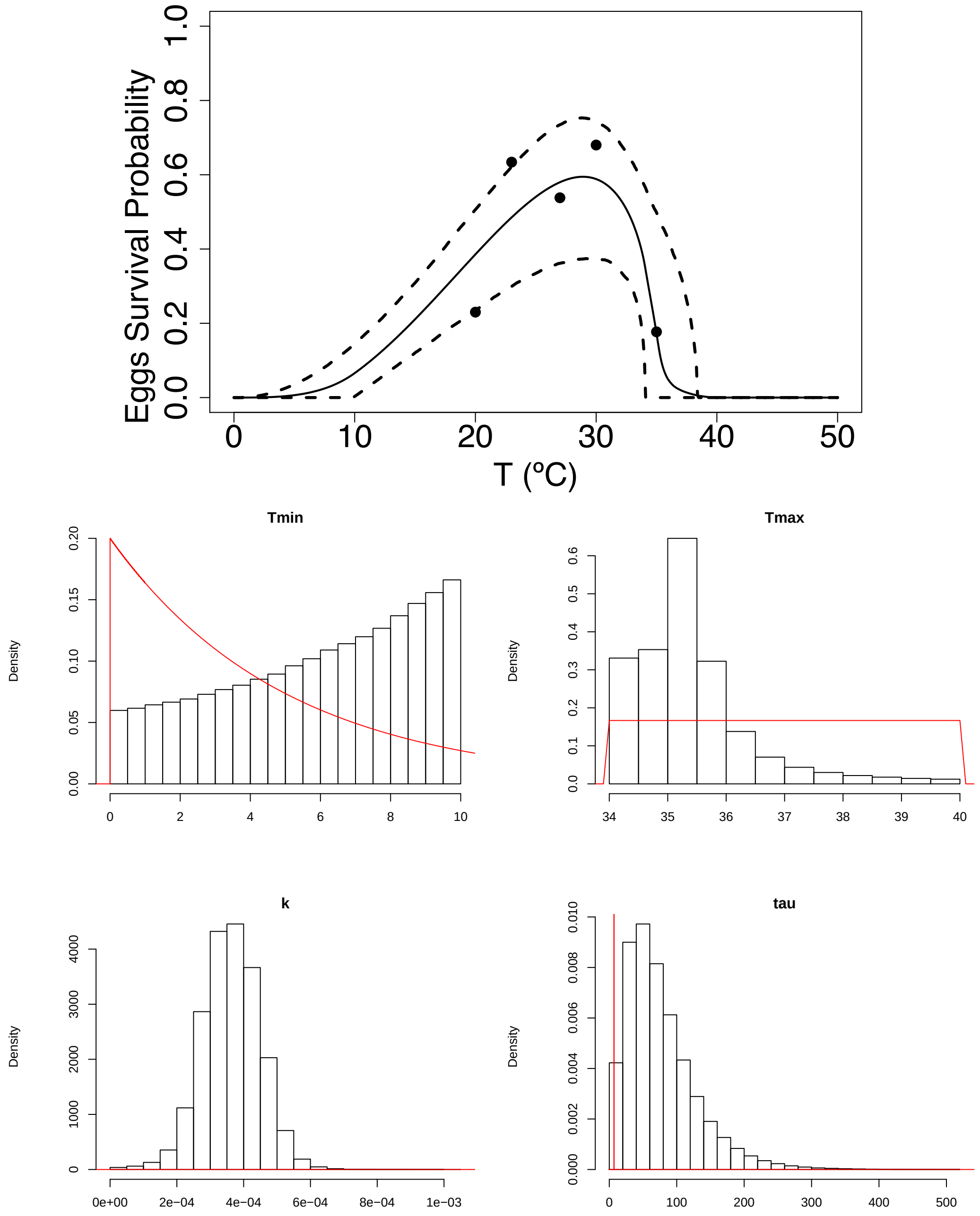

Figure A.5: (Top) The mean trajectory in solid line and HPD interval in dashed black for the egg survival probability p_E. (Bottom) Histograms of the posterior distribution for each parameter of the Brière fit for the probability p_E. The prior distribution for each parameter is plotted in red. The Brière fit is determined by the equation $kT(T - T_{Min})\sqrt{T_{Max} - T}$ using a normal distribution with precision τ.

Adult mortality rate μ

The rate at which midges die over a span of time is known as the mortality rate μ. We define the mortality rate of midges as $\frac{1}{lf}$, where lf represents the lifespan of midges in days,

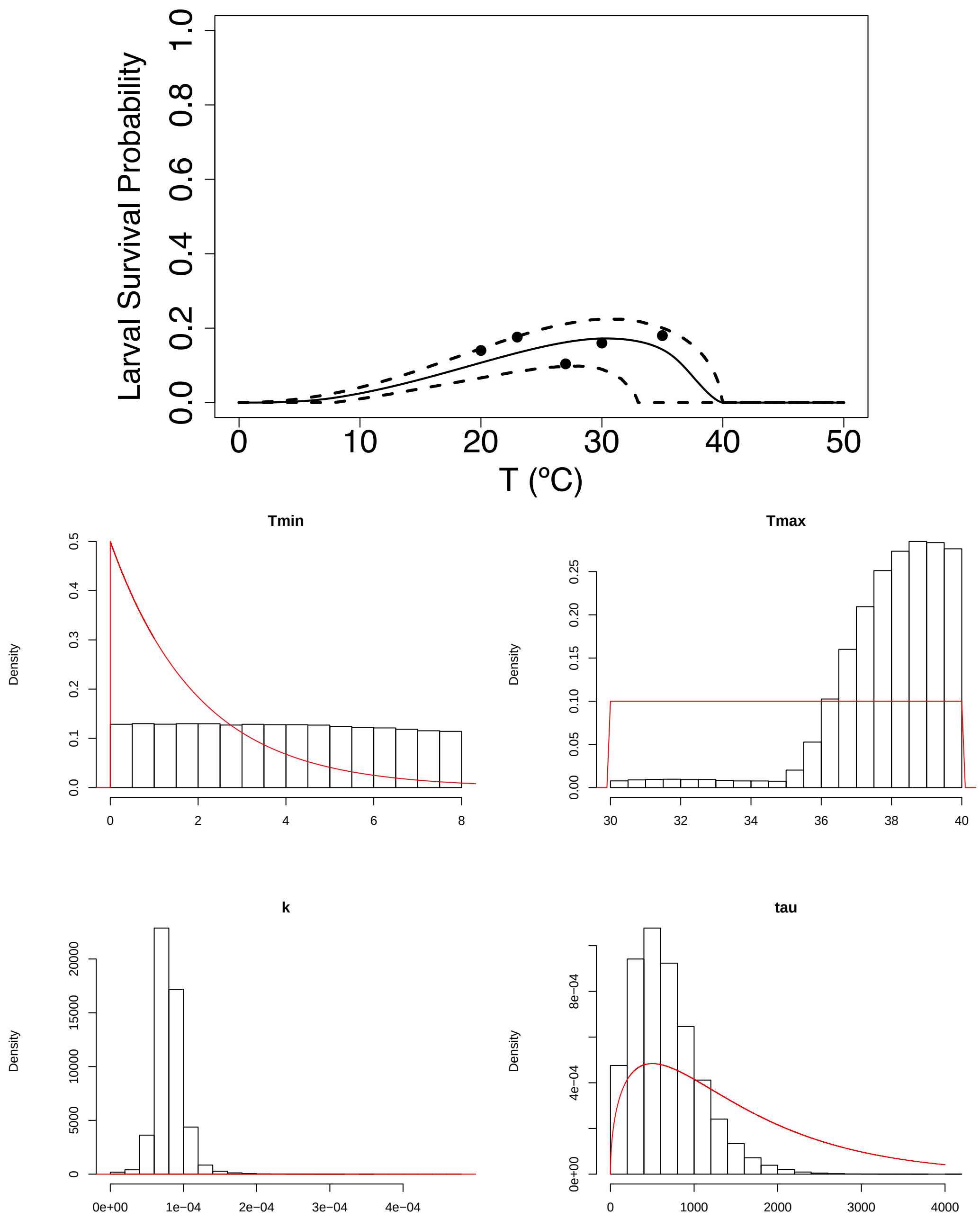

Figure A.6: (Top) The mean trajectory in solid line and HPD interval in dashed black for the larval survival probability p_L. (Bottom) Histograms of the posterior distribution for each parameter of the Brière fit for the probability p_L. The prior distribution for each parameter is plotted in red. The Brière fit is determined by the equation $kT(T - T_{Min})\sqrt{T_{Max} - T}$ using a normal distribution with precision τ.

or the probability of survival for the midges. We define mortality rate in the case where lf is the lifespan of midges in days. Mortality rate is also sensitive to environmental factors, especially temperature [3].

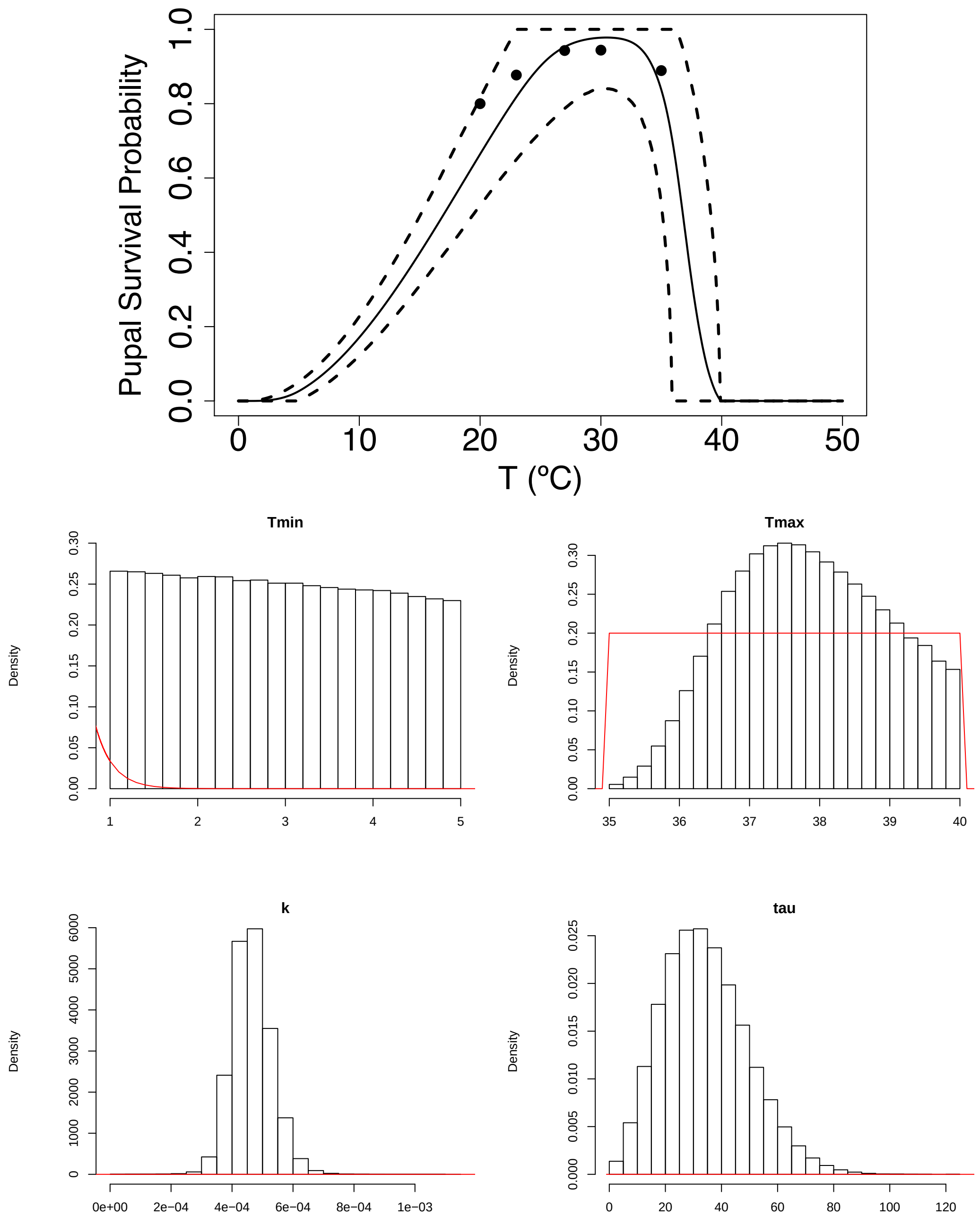

Figure A.7: (Top) The mean trajectory in solid line and HPD interval in dashed black for the pupal survival probability p_P. (Bottom) Histograms of the posterior distribution for each parameter of the Brière fit for the probability p_P. The prior distribution for each parameter is plotted in red. The Brière fit is determined by the equation $kT(T - T_{Min})\sqrt{T_{Max} - T}$ using a normal distribution with precision τ.

Thermal traits prior distributions

Table A.2 summarizes all the priors used to fit the thermal curves.

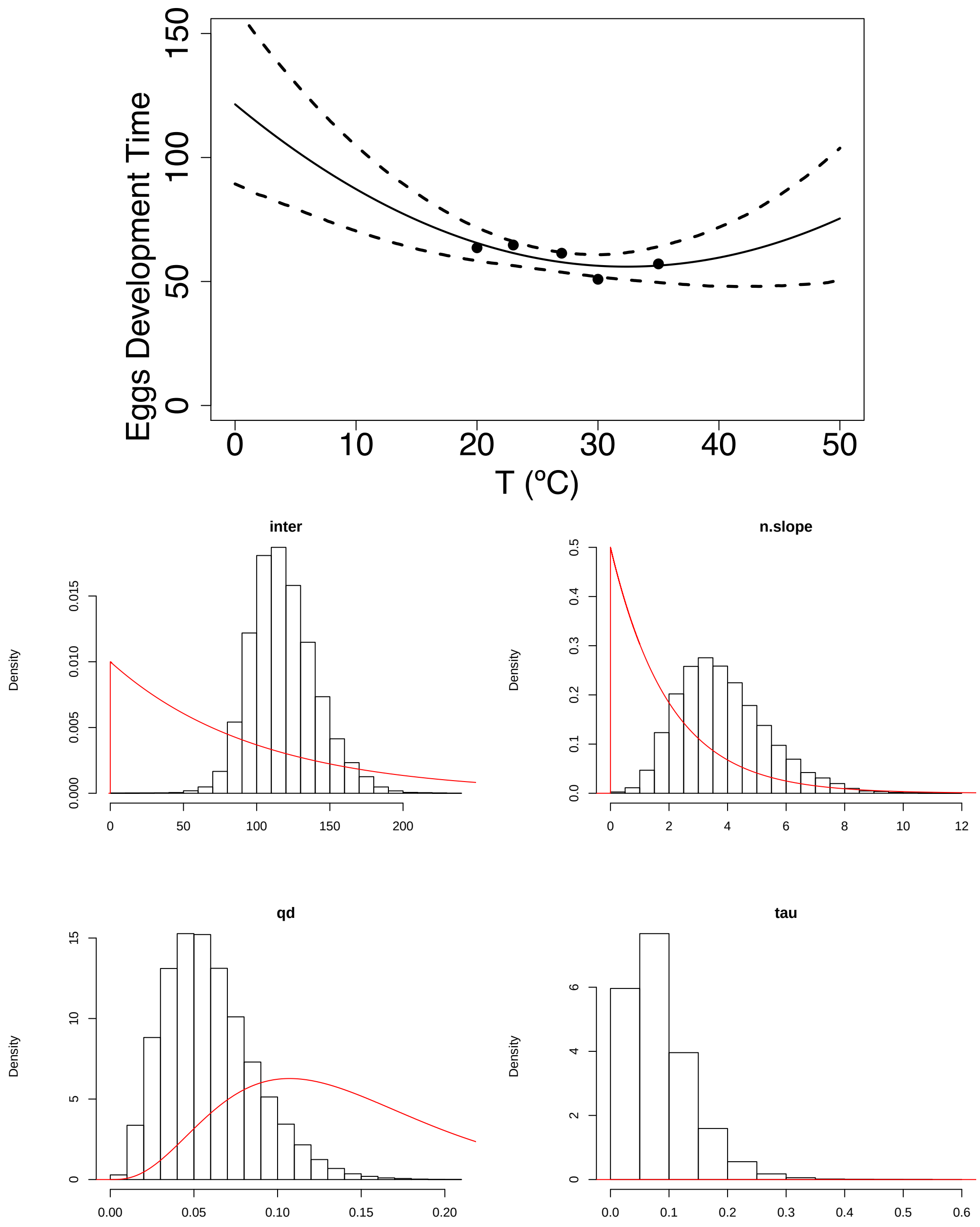

Figure A.8: (Top) The mean trajectory in solid line and HPD interval in dashed black for egg development time ρ_E. (Bottom) Histograms of the posterior distribution for each parameter of the quadratic fit for egg development time ρ_E. The prior distribution for each parameter is plotted in red. The quadratic fit is determined by the equation $inter - n.slope\, T + qd\, T^2$ using a normal distribution with precision τ.

A.5 Posterior distributions for all $S(T)$ forms

For all three R_0 posterior distributions we provide posterior distributions for the lower temperature limit, peak temperature, and upper temperature limit.

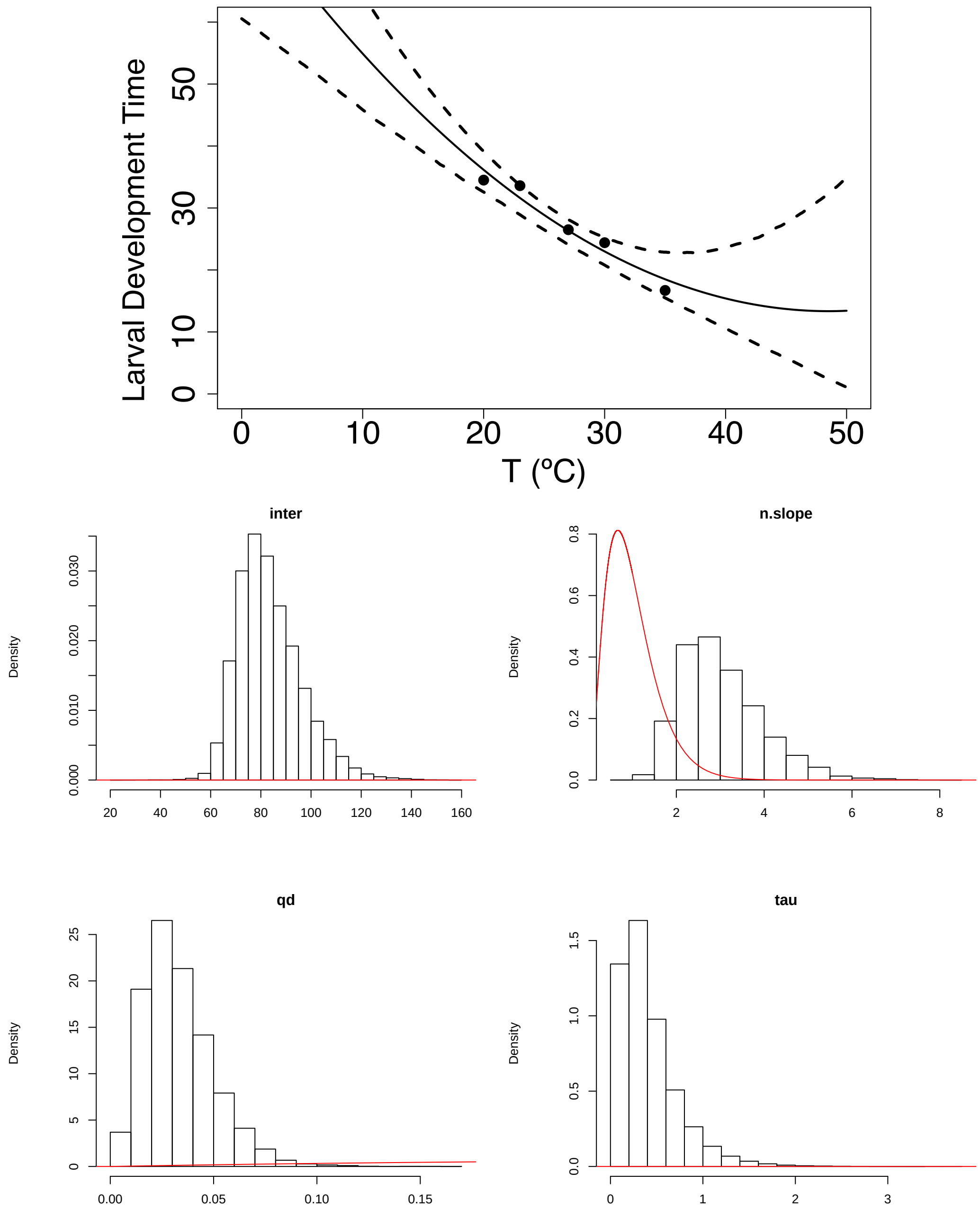

Figure A.9: (Top) The mean trajectory in solid line and HPD interval in dashed black for larval development time ρ_L. (Bottom) Histograms of the posterior distribution for each parameter of the quadratic fit for larval development time ρ_L. The prior distribution for each parameter is plotted in red. The quadratic fit is determined by the equation $inter - n.slope\, T + qd\, T^2$ using a normal distribution with precision τ.

A.6 Digitized data

Table A.3 shows the digitized trait values and their corresponding references.

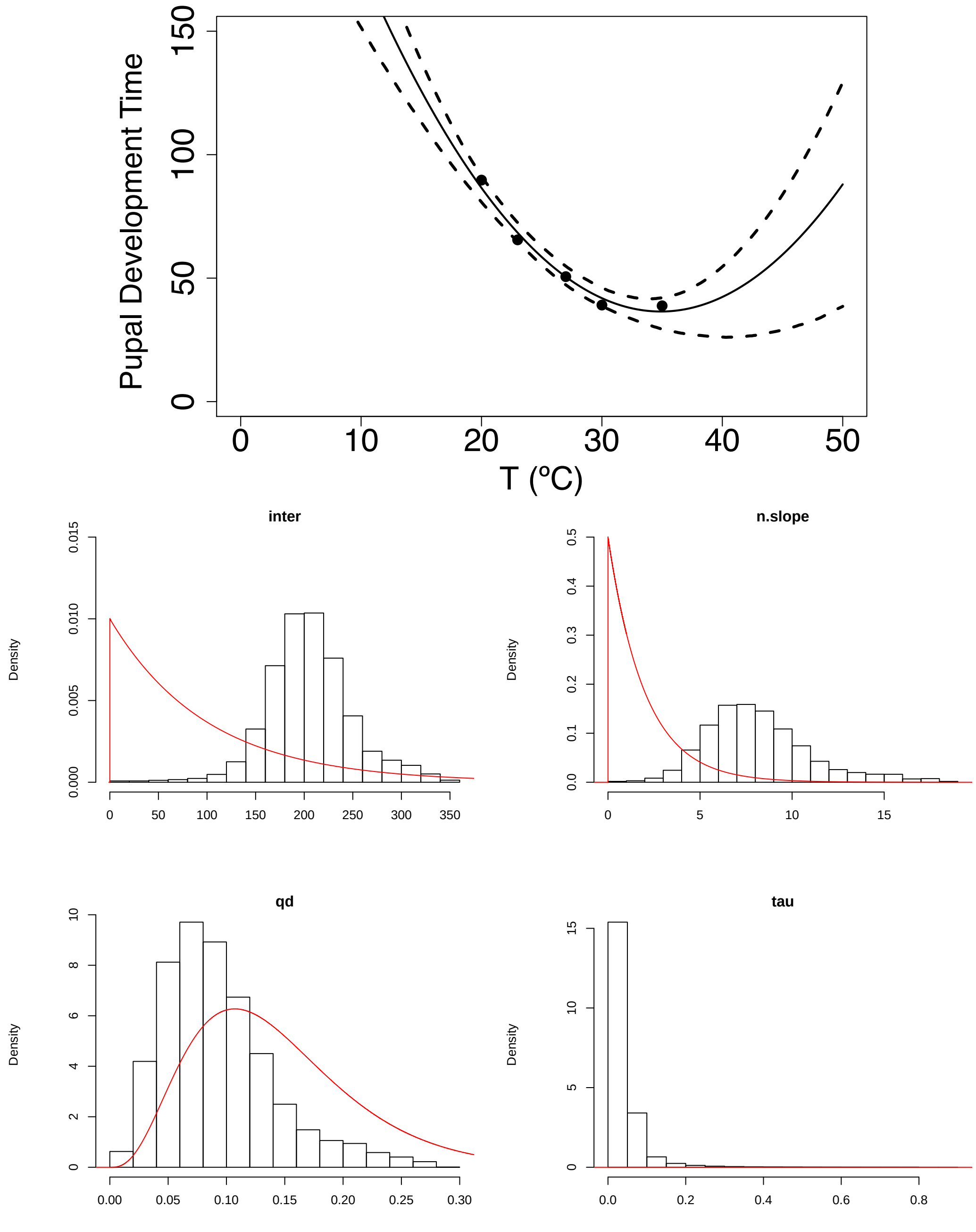

Figure A.10: (Top) The mean trajectory in solid line and HPD interval in dashed black for pupal development time ρ_P. (Bottom) Histograms of the posterior distribution for each parameter of the quadratic fit for pupal development time ρ_P. The prior distribution for each parameter is plotted in red. The quadratic fit is determined by the equation $inter - n.slope\, T + qd\, T^2$ using a normal distribution with precision τ.

Parameter	Trait	Value	Units	Transformed	Ref.
a	Vector biting rate	0.05	bites/day	Y	48

		0.03<br>0.08<br>0.18<br>0.29<br>0.4<br>0.4<br>0.28<br>0.28<br>0.66<br>0.66<br>0.08<br>0.08<br>0.15<br>0.19<br>0.66			
b	Probability of transmission	0.08<br>0.28<br>0.28<br>0.88<br>0.96	dimensionless	Y	50
efd	Fecundity	5.528<br>3.122<br>13.11<br>9.745<br>6.206<br>31.191<br>19.034	# eggs per female per day	Y	48

		1.361			
		1.242			
		11.08			
		13.961			
		17.93			
		41.531			
		60.535			
		39.856			
		51.724			
		69.951			
		12.731			
		1.154			
		36.417			
		0.465			
		5.844			
		7.048			
		19.469			
		31.938			
		21.195			
		12.255			
		22.332			
		0.365			
		1.703			
		1.536			
edt	Egg's development time	63.6	Days	N	51
		64.7			
		61.4			

		50.9			
		57.1			
ldt	Larva's development time	34.5	Days	N	51
		33.6			
		26.5			
		24.4			
		16.7			
$PuDt$	Pupa's development time	89.7	Days	N	51
		65.5			
		50.6			
		39.1			
		38.8			
μ	Adult's mortality	0.037	$\frac{1}{\text{Days}}$	Y	48
	rate	0.057			
		0.072			
		0.057			
		0.121			
		0.058			
		0.078			
		0.084			
		0.067			
		0.073			
		0.045			
		0.056			
		0.077			
		0.068			
		0.079			

		0.114			
		0.138			
		0.073			
		0.078			
		0.113			
pdr	Extrinsic incubation period (EIP)	0.051	$\frac{1}{\text{Days}}$	Y	52
		0.04			
		0.021			
		0.052			
		0.08			
		0.073			
		0.073			
		0.073			
		0.073			
		0.069			
		0.101			
		0.101			
		0.14			
		0.143			
		0.144			
		0.208			
		0.2			
		0.248			
pE	Egg's survival probability	0.23	dimensionless	N	51
		0.634			
		0.538			
		0.68			

		0.177			
pL	Larva's survival probability	0.14<br>0.176<br>0.104<br>0.16<br>0.18	dimensionless	N	51
pP	Pupa's survival Probability	0.8<br>0.877<br>0.943<br>0.944<br>0.889	dimensionless	N	51

Table A.3: Traait values digitized and fit using MCMC.

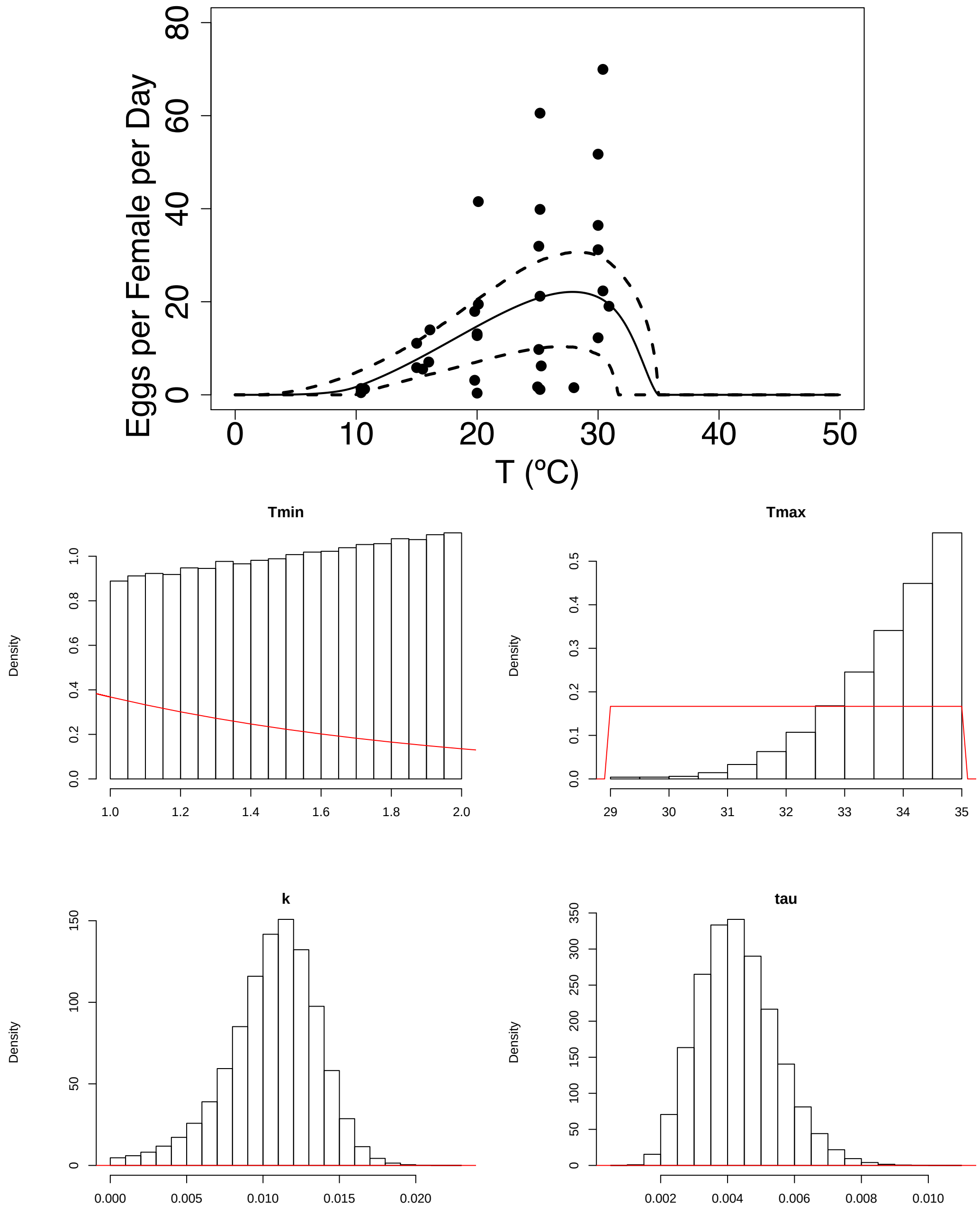

Figure A.11: (Top) The mean trajectory in solid line and HPD interval in dashed black for fecundity F. (Bottom) Histograms of the posterior distribution for each parameter of the Brière fit for fecundity F. The prior distribution for each parameter is plotted in red. The Brière fit is determined by the equation $kT(T - T_{Min})\sqrt{T_{Max} - T}$ using a normal distribution with precision τ.

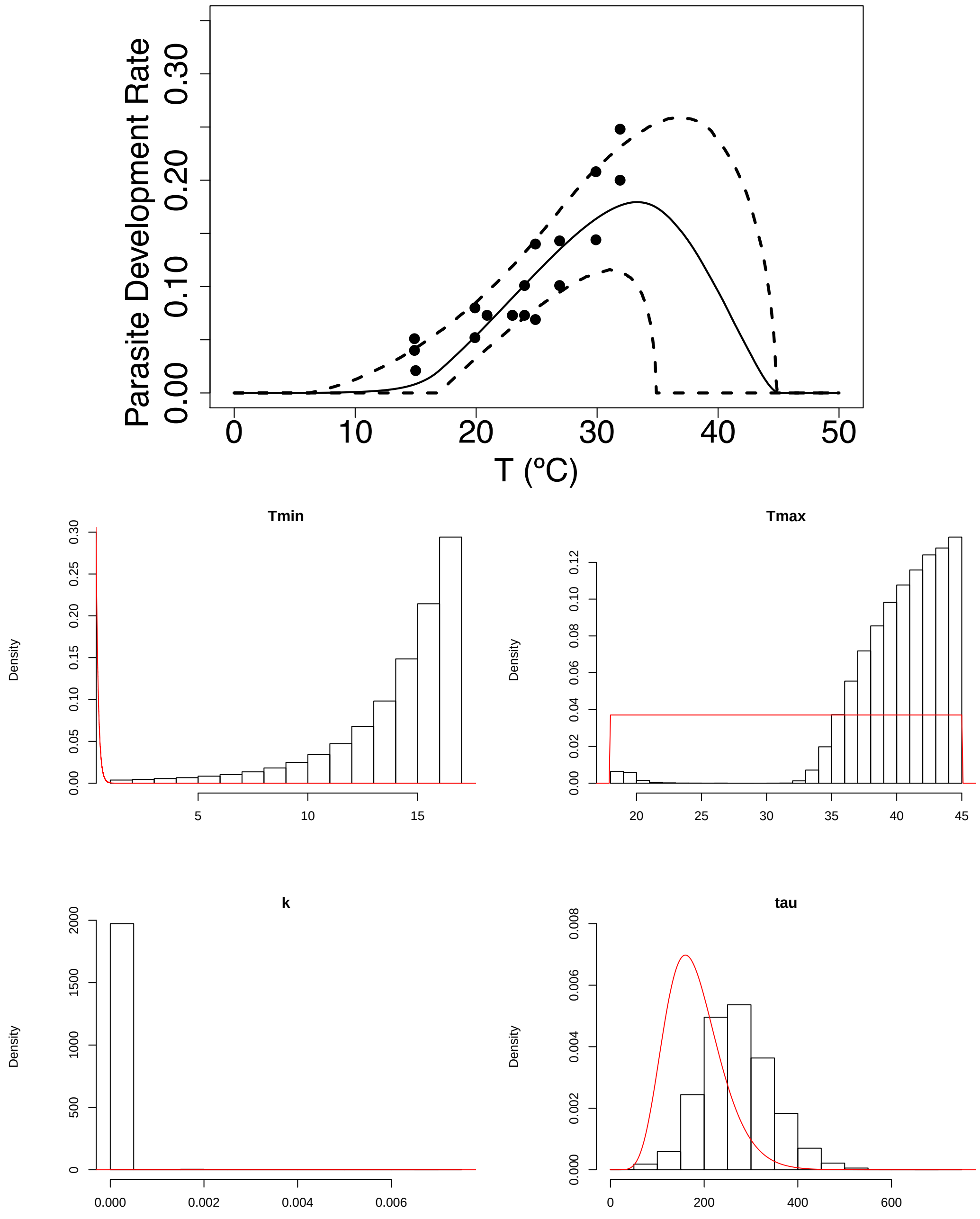

Figure A.12: (Top) The mean trajectory in solid line and HPD interval in dashed black for the parasite development rate ν. (Bottom) Histograms of the posterior distribution for each parameter of the Brière fit for the parasite development rate ν. The prior distribution for each parameter is plotted in red. The Brière fit is determined by the equation $kT(T - T_{Min})\sqrt{T_{Max} - T}$ using a normal distribution with precision τ.

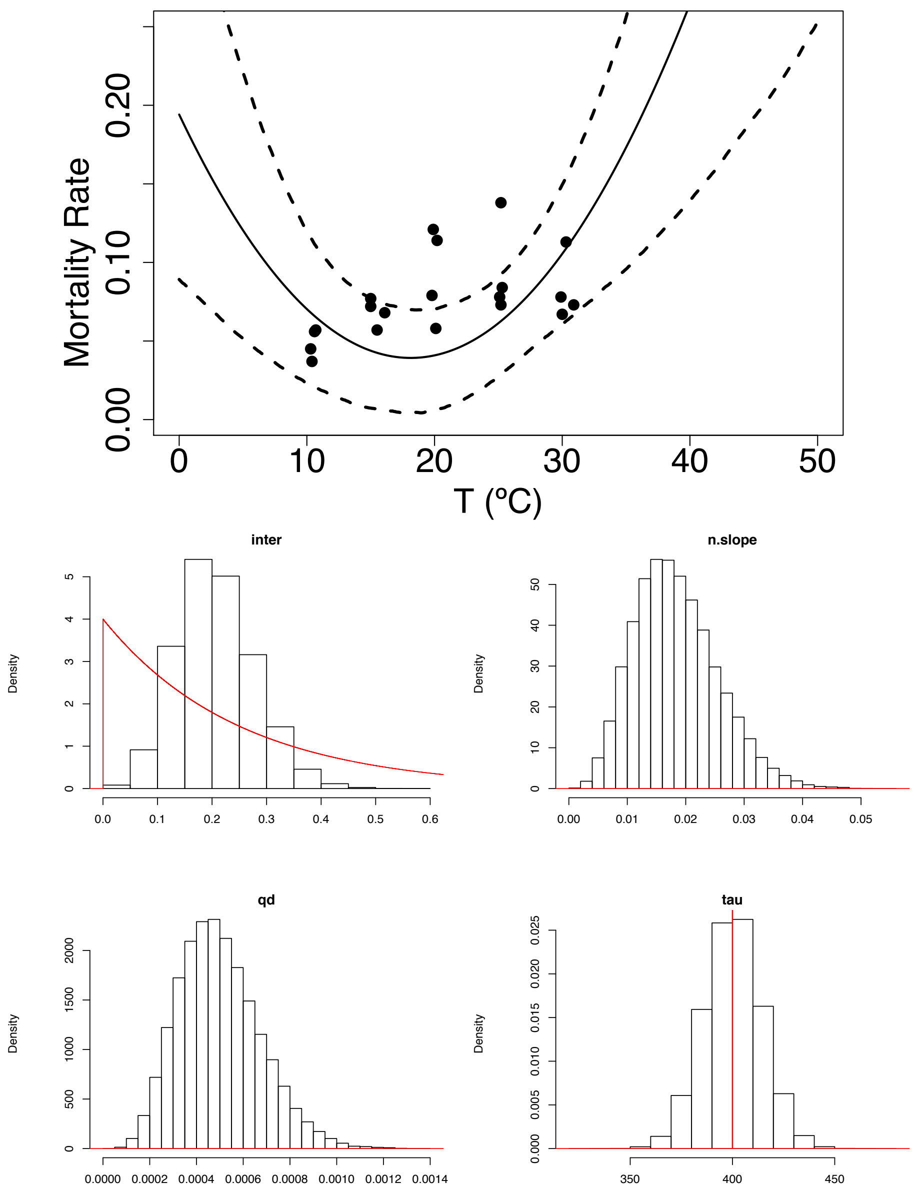

Figure A.13: (Top) The mean trajectory in solid line and HPD interval in dashed black for the mortality rate μ. (Bottom) Histograms of the posterior distribution for each parameter of the quadratic fit for the mortality rate μ. The prior distribution for each parameter is plotted in red. The quadratic fit is determined by the equation $inter - n.slope\, T + qd\, T^2$ using a normal distribution with precision τ.

Model Parameter	Mean Function	Parameters	Prior
Biting Rate<br>a	Brière	T_{Min}<br>T_{Max}<br>k<br>τ	dunif(0, 20)<br>dunif(20,40)<br>dgamma(1,20)<br>dgamma(0.01, 0.01)
Transmission probability<br>b	Brière	T_{Min}<br>T_{Max}<br>k	dunif(10,24)<br>dunif(25,35)<br>dgamma(1,10)
Egg Survival Probability<br>p_E	Brière	T_{Min}<br>T_{Max}<br>k<br>τ	dunif(10,20)<br>dunif(35,40)<br>dgamma(1,20)<br>dgamma(7, 5^{-10})
Larval Survival Probability<br>p_L	Brière	T_{Min}<br>T_{Max}<br>k<br>τ	dunif(0,8)<br>dunif(30,40)<br>dgamma(1,20)<br>dgamma(1.5, 0.001)
Pupal Survival Probability<br>p_P	Brière	T_{Min}<br>T_{Max}<br>k<br>τ	dunif(1,5)<br>dunif(35,40)<br>dgamma(1,5)<br>dgamma(10, 0.002)
Egg Development Time<br>ρ_E	Quadratic	inter<br>n.slope<br>qd<br>τ	dgamma(1, 0.01)<br>dgamma(1, 0.5)<br>dgamma(4,28)<br>dnorm(3, 1/800)
Larval Development Time<br>ρ_L	Quadratic	inter<br>n.slope<br>qd<br>τ	dgamma(1, 0.01)<br>dgamma(1, 0.5)<br>dgamma(4,28)<br>dnorm(3, 1/1000)
Pupal Development Time<br>ρ_P	Quadratic	inter<br>n.slope<br>qd<br>τ	dgamma(1, 0.01)<br>dgamma(1, 0.5)<br>dgamma(4,28)<br>dnorm(3, 1/200)
Eggs per Female per Day<br>F	Brière	T_{Min}<br>T_{Max}<br>k<br>τ	dunif(1, 10)<br>dunif(29,35)<br>dgamma(1,1)<br>dgamma(9, 0.0005)
Parasite Development Rate<br>ν	Brière	T_{Min}<br>T_{Max}<br>k<br>τ	dunif(1, 17)<br>dunif(18,45)<br>dgamma(1,10)<br>dgamma(9, 0.05)
Adult Mortality Rate<br>μ	Quadratic	inter<br>n.slope<br>qd<br>τ	dgamma(2,2)<br>dgamma(3,3)<br>dgamma(2,2)<br>dnorm(1000, 1/500)

Table A.2: Prior distributions for each of the parameters for the fitting of the responses for each of the thermal traits considered.

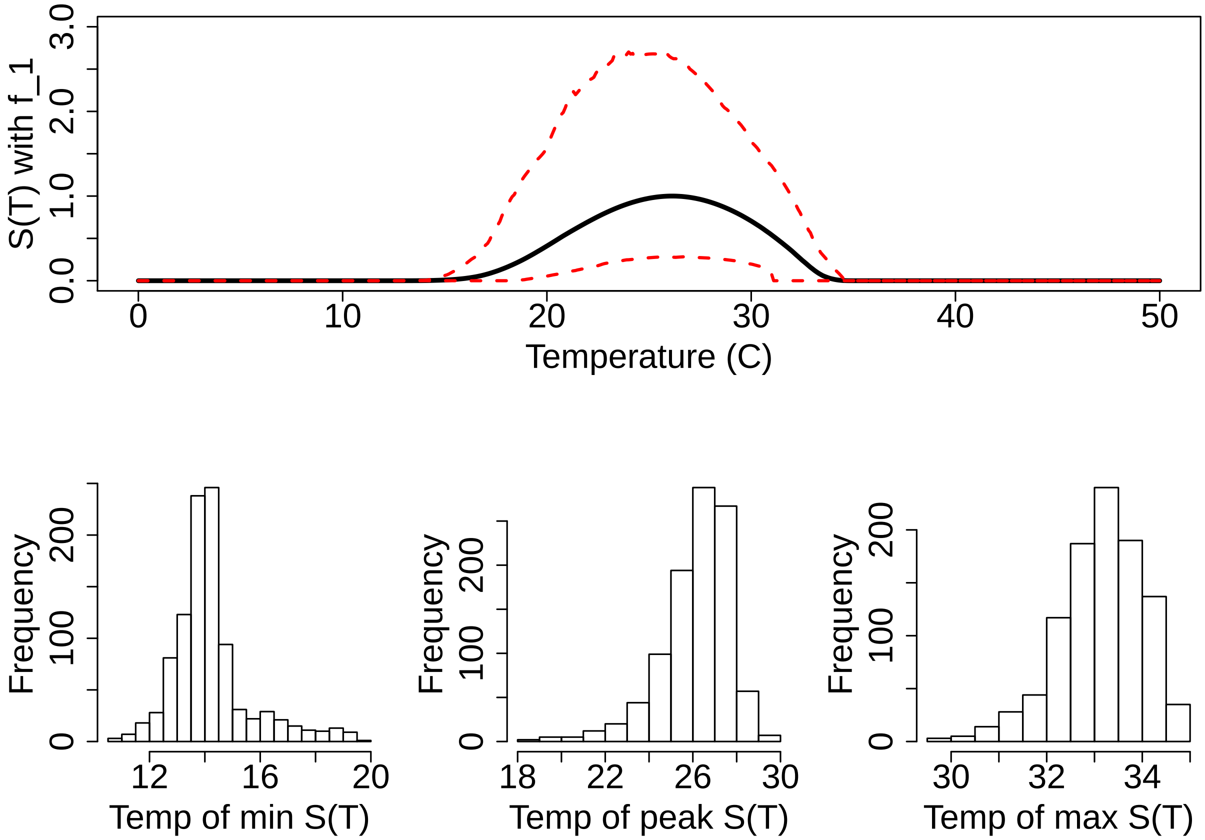

Figure A.14: Minimum, peak and, maximum temperatures posterior densities for Dietz 1993 [8] R_0

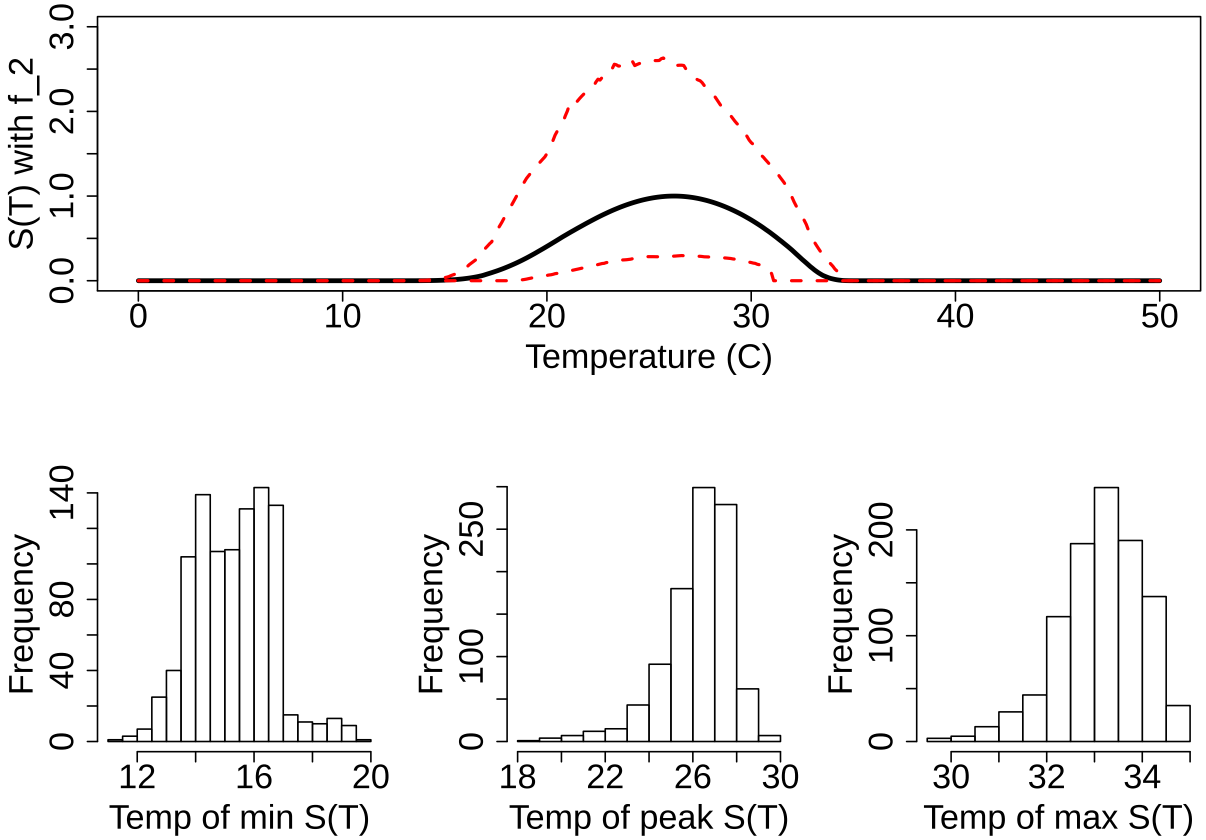

Figure A.15: Minimum, peak and, maximum temperatures posterior densities for Gubbins 2008 [9] R_0

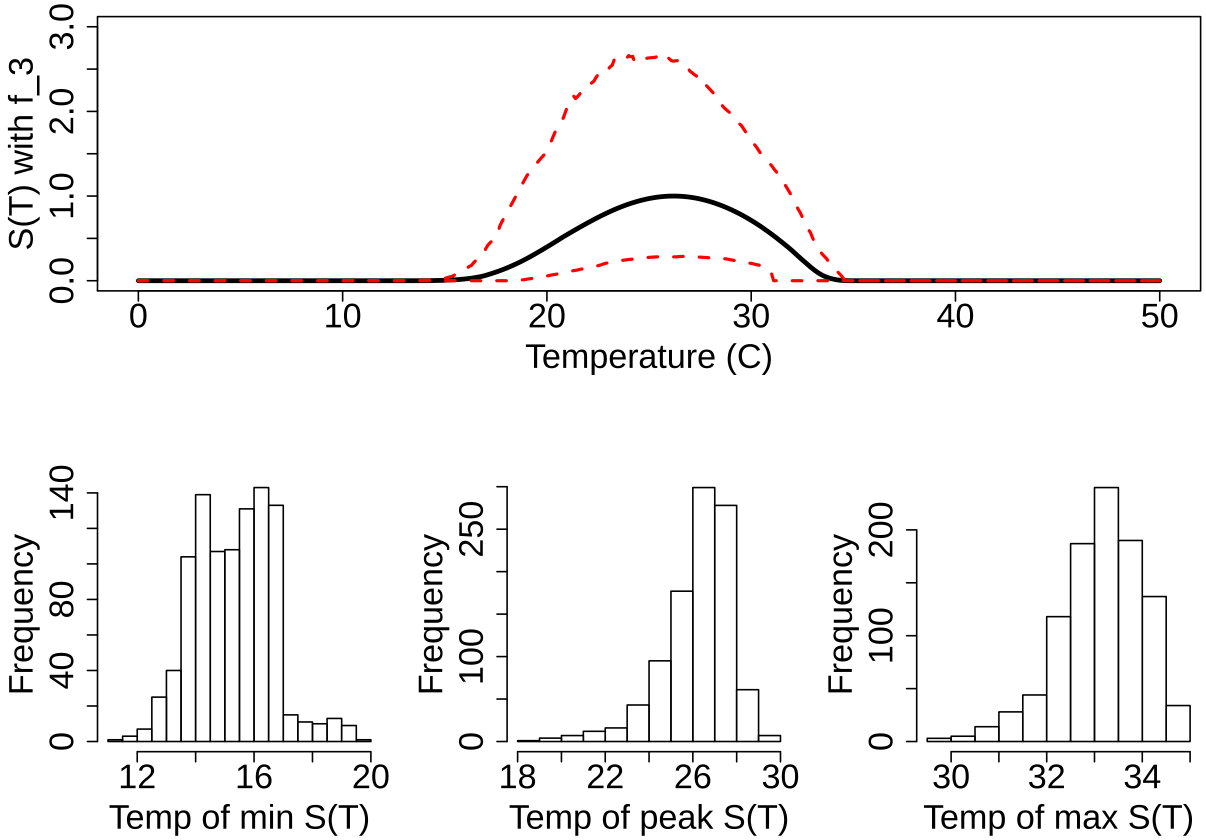

Figure A.16: Minimum, peak and, maximum temperatures posterior densities for the R_0 presented here.

References

[1] A. L. Lloyd, "Realistic distributions of infectious periods in epidemic models: changing patterns of persistence and dynamics," *Theoretical Population Biology*, vol. 60, no. 1, pp. 59–71, 2001.

[2] P. E. Parham and E. Michael, "Modeling the effects of weather and climate change on malaria transmission," *Environmental Health Perspectives*, vol. 118, no. 5, p. 620, 2010.

[3] T. J. Lysyk and T. Danyk, "Effect of temperature on life history parameters of adult Culicoides sonorensis (Diptera: Ceratopogonidae) in relation to geographic origin and vectorial capacity for bluetongue virus," *Journal of Medical Entomology*, vol. 44, no. 5, pp. 741–751, 2007.

[4] B. Mullens, A. Gerry, T. Lysyk, and E. Schmidtmann, "Environmental effects on vector competence and virogenesis of bluetongue virus in Culicoides: interpreting laboratory data in a field context," *Vet Ital*, vol. 40, no. 3, pp. 160–166, 2004.

[5] S. Carpenter, A. Wilson, J. Barber, E. Veronesi, P. Mellor, G. Venter, and S. Gubbins, "Temperature dependence of the extrinsic incubation period of orbiviruses in Culicoides biting midges," *PloS one*, vol. 6, no. 11, p. e27987, 2011.

[6] J. Vaughan and E. Turner Jr, "Development of immature Culicoides variipennis (Diptera: Ceratopogonidae) from Saltville, Virginia, at constant laboratory temperatures," *Journal of medical entomology*, vol. 24, no. 3, pp. 390–395, 1987.

[7] C. D. Harvell, C. E. Mitchell, J. R. Ward, S. Altizer, A. P. Dobson, R. S. Ostfeld, and M. D. Samuel, "Climate warming and disease risks for terrestrial and marine biota," *Science*, vol. 296, no. 5576, pp. 2158–2162, 2002.

[8] K. Dietz, "The estimation of the basic reproduction number for infectious diseases," *Statistical methods in medical research*, vol. 2, no. 1, pp. 23–41, 1993.

[9] S. Gubbins, S. Carpenter, M. Baylis, J. L. Wood, and P. S. Mellor, "Assessing the risk of bluetongue to UK livestock: uncertainty and sensitivity analyses of a temperature-dependent model for the basic reproduction number," *Journal of the Royal Society Interface*, vol. 5, no. 20, pp. 363–371, 2007.
